# Distannabarrelenes with Three Coordinated Sn^II^ Atoms

**DOI:** 10.1002/chem.202001432

**Published:** 2020-07-28

**Authors:** Mahendra K. Sharma, Timo Glodde, Beate Neumann, Hans‐Georg Stammler, Rajendra S. Ghadwal

**Affiliations:** ^1^ Molecular Inorganic Chemistry and Catalysis, Inorganic and Structural Chemistry, Center for Molecular Materials Faculty of Chemistry Universität Bielefeld Universitätsstrasse 25 33615 Bielefeld Germany

**Keywords:** barrelenes, ditopic carbenes, mixed valency, stannylenes, tin

## Abstract

Crystalline 1,4‐distannabarrelene compounds [(ADC^Ar^)_3_Sn_2_]SnCl_3_ (**3**‐**Ar**) (ADC^Ar^={ArC(NDipp)_2_CC}; Dipp=2,6‐*i*Pr_2_C_6_H_3_, Ar=Ph or DMP; DMP=4‐Me_2_NC_6_H_4_) derived from anionic dicarbenes Li(ADC^Ar^) (**2**‐**Ar**) (Ar=Ph or DMP) have been reported. The cationic moiety of **3**‐**Ar** features a barrelene framework with three coordinated Sn^II^ atoms at the 1,4‐positions, whereas the anionic unit SnCl_3_ is formally derived from SnCl_2_ and chloride ion. The all carbon substituted bis‐stannylenes **3**‐**Ar** have been characterized by NMR spectroscopy and X‐ray diffraction. DFT calculations reveal that the HOMO of **3**‐**Ph** (*ϵ*=−6.40 eV) is mainly the lone‐pair orbital at the Sn^II^ atoms of the barrelene unit. **3**‐**Ar** readily react with sulfur and selenium to afford the mixed‐valence Sn^II^/Sn^IV^ compounds [(ADC^Ar^)_3_SnSn(E)](SnCl_6_)_0.5_ (E=S **4**‐**Ar**, Ar=Ph or DMP; E=Se **5**‐**Ph**).

## Graphical abstract

**Crystalline 1,4‐distannabarrelene compounds** [(ADC^Ar^)_3_Sn_2_]SnCl_3_ (**3‐Ar**) (ADC^Ar^={ArC(NDipp)_2_CC}; Dipp=2,6‐*i*Pr_2_C_6_H_3_, Ar=Ph or DMP; DMP=4‐Me_2_NC_6_H_4_)) have been reported. The cationic moiety of **3‐Ar** features a barrelene framework with three coordinated Sn^II^ atoms at the 1,4‐positions, whereas the anionic unit SnCl_3_^−^ is formally derived from SnCl_2_ and chloride ion.

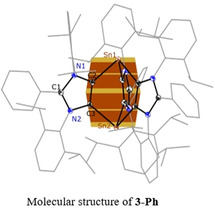

Exploration of compounds featuring a low‐valent main‐group element(s) has been a fascinating research topic in fundamental chemistry because of their intriguing electronic structure^1^ and reactivity.^2^ Heavier main‐group element compounds that are analogues to ubiquitous organic molecules such as alkenes, alkynes, and other unsaturated species have been appealing synthetic targets.^3^ Barrelene, bicyclo[2.2.2]octa‐2,5,7‐triene (**I**) (Figure 1) is the formal Diels–Alder adduct of acetylene **II** and benzene **III**.^4^ The name “barrelene” was coined because of its barrel like shape (Figure 1). Barrelene first caught attention in 1955 when Hine et al. noted that this molecule might be aromatic.^5^ Since the first synthesis of **I** by Zimmerman and Paufler in 1960,^4^ this intriguing molecule has been in focus of synthetic as well as theoretical chemists.^6^ Involvement of barrelene type species have also been predicated in the activation of organic substrates with low‐valent main group compounds.^7^

**Figure 1:**
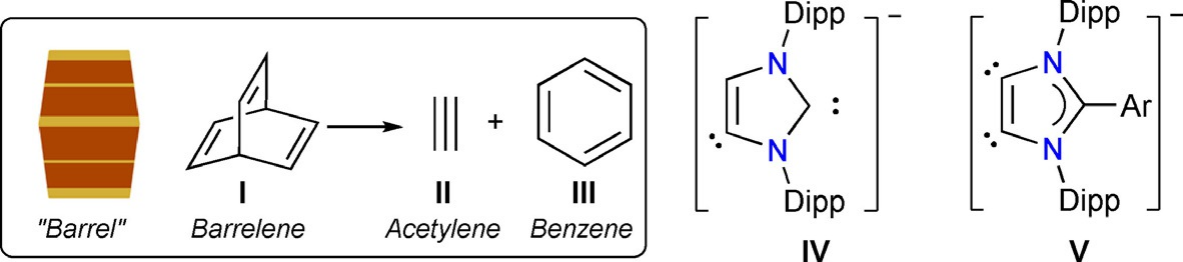
Barrelene **I**, acetylene **II**, benzene **III**, C2/C4‐anionic dicarbene (ADC) **IV**, and C4/C5 ADC **V**.

Some barrelene type compounds featuring a Group 13 or 15 element(s) have been isolated over the past years,^8^ however, related species featuring Group 14 elements (tetreles), the heavier carbon congeners, remained scarce. The first silabarrelene was reported in 1977 by Barton and Banasiak,^9^ which was prepared by the Diels–Alder reaction of an in situ generated silabenzene with an alkyne. Synthesis of barrelene derivatives containing heavier Group 14 elements by classical cycloaddition reactions seems a demanding task because of the synthetic inaccessibility of suitable unsaturated precursors.^10^ Breher^11^ and Stalke^12^ independently reported barrelene type compounds featuring Ge^II^ or Sn^II^ atoms using pyrazole frameworks, showing an alternative way to access these species, in which the bicyclo[2.2.2] framework is based on nitrogen instead of carbon atoms. Subsequently, several other main‐group element systems based pyrazole scaffolds have been also reported.^13^

Robinson et al. reported the C4‐H deprotonation of an N‐heterocyclic carbene (NHC), the IPr (IPr=C{(NDipp)CH}_2_, Dipp=2,6‐*i*Pr_2_C_6_H_3_), with *n*BuLi to access an anionic dicarbene (ADC) **IV** (Figure 1).^14^ Over the past years, this and related species have been extensively explored by Goicoechea, Mulvey, Hevia, and other research groups in main‐group as well as in transition metal chemistry.^15^ The C2/C4‐positions of **IV** are remotely located and thus are not suitable for the preparation of cyclic compounds. We recently reported ADCs **V** that feature carbenes at the vicinal C4/C5‐positions^16^ and hence should be an appropriate choice for constructing heterocyclic rings containing heavier main‐group elements.^17^ Herein, we report the first distannabarrelenes [(ADC^Ar^)_3_Sn_2_]SnCl_3_ (ADC^Ar^=ArC(NDipp)_2_CC; Ar=Ph, **3‐Ph**; DMP, **3‐DMP**; DMP=4‐Me_2_NC_6_H_4_) featuring three‐coordinated tin(II) atoms as crystalline solids and describe their structure and reactivity (Scheme 1).

**Scheme 1:**
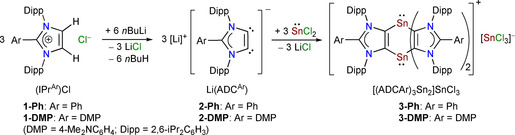
Synthesis of distannabarrelene compounds **3‐Ph** and **3‐DMP**.

The anionic dicarbenes Li(ADC^Ar^) (Ar=Ph, **2‐Ph**; DMP, **2‐DMP**) are readily accessible by the double deprotonation of C2‐arylated 1,3‐imidazolium salts (IPr^Ar^)Cl (IPr^Ar^=ArC{(NDipp)CH}_2_; Ar=Ph, **1‐Ph**; DMP, **1‐DMP**; Dipp=2,6‐*i*Pr_2_C_6_H_3_) with *n*BuLi.^16^ Treatment of freshly prepared **2‐Ph** and **2‐DMP** with SnCl_2_ affords the compounds [(ADC^Ar^)_3_Sn_2_]SnCl_3_ (Ar=Ph, **3‐Ph** (76 %); DMP, **3‐DMP** (95 %)) (Scheme 1). **3‐Ph** and **3‐DMP** are ionic species, each comprising a cationic 1,4‐distannabarallene and an anionic SnCl_3_ moieties. The ADC^Ar^ moiety in **3‐Ar** serves as a mono‐anionic four‐electron donor and the remaining chloride combines with an additional SnCl_2_ to form the SnCl_3_ counter anion.^18^ **3‐Ph** and **3‐DMP** are colorless crystalline solids and are stable both in solution as well as in the solid state under an inert gas atmosphere.

The ^1^H NMR spectra of **3‐Ph** and **3‐DMP** each shows four doublets and two septets for the isopropyl groups. The ^13^C{^1^H} NMR spectra of **3‐Ph** and **3‐DMP** exhibit well resolved resonances for the ADC^Ar^ unit, which are consistent with their ^1^H NMR signals. The ^119^Sn{^1^H} NMR spectrum of **3‐Ph** (−298.6 ppm) and **3‐DMP** (−297.5 ppm) each shows a singlet, indicating that both the tin atoms of the cationic part are magnetically equivalent. The ^119^Sn{^1^H} NMR signals of **3‐Ar** are high‐field shifted compared to those of (NHC)SnX_2_ (X=Si(SiMe_3_)_3_ −196.8 ppm; Ge(SiMe_3_)_3_ −115 ppm; 2,6‐(2,4,6‐*i*Pr_3_C_6_H_2_)C_6_H_3_ −150 ppm, Ph −121 ppm)^19^ that is consistent with the stronger σ‐donor property of ADCs **2‐Ar** compared to classical NHCs.^20^ They are, however, downfield shifted with respect to that of the poly(pyrazolyl)stannylenes [Sn_2_(3,5‐Me_2_Pz)_3_][SnCl_3_] (−337 and −498 ppm)^12^ and [{Sn(3,5‐R_2_Pz)_2_}_2_] (R=CF_3_, CMe_3_) (−720 ppm).^11^

The solid‐state molecular structures^21^ of **3‐Ph** and **3‐DMP** (Figure 2) show three‐fold coordinated tin atoms at the apexes of a cationic bicyclo[2.2.2] framework along with [SnCl_3_^−^] or a mixture of [SnCl_3_^−^] and chloride (in ratio 79:21) as a counter anion, respectively. Each of the tin atoms features a trigonal pyramidal geometry and binds to the backbone carbon atoms of three ADC^Ar^ and comprises one stereoactive electron lone pair. The Sn−C_ADC_ bond lengths (2.24 to 2.27 Å) of **3‐Ar** (Table 1) are comparable with the Sn−C bond length of Goicoechea's C4‐bound Sn^II^‐NHC compound (2.248(4) Å),15j but are slightly smaller than that of Jones's Sn^0^ [(IPr)_2_Sn_2_ 2.297 Å]^22^ and Rivard's Sn^II^ [(IPr)SnCl_2_ 2.341(8) Å]^23^ compounds. The C_ADC_−Sn−C_ADC_ bond angles in **3‐Ar** (86.9 to 90.1°) are in line with those of the N‐Sn‐N bond angles (85.1 to 92.0°) of the poly(pyrazolyl)stannylenes [Sn_2_(3,5‐Me_2_Pz)_3_][SnCl_3_] consisting six nitrogen atoms on a paddle‐wheel with two Sn^II^ atoms on the shaft.^12^ The *trans*‐annular Sn–Sn distance in **3‐Ar** is ca. 4.0 Å.

**Figure 2:**
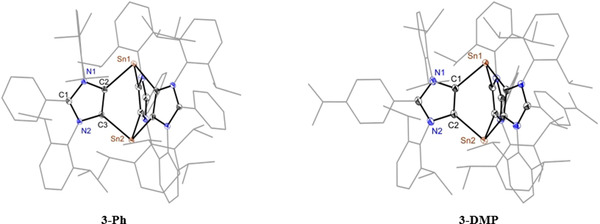
Solid‐state molecular structures of **3‐Ph** and **3‐DMP**. Hydrogen atoms and the counter anion (SnCl_3_^−^) are omitted and aryl groups are shown as wireframe models for clarity.

**Table 1:** Selected bond lengths (Å) and angles (°): for **3‐Ar**, **4‐Ph**, and **5‐Ph**.

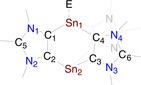						
Compound	Sn1−C1 Sn2−C2	C1−C2 C3−C4	C1−N1 C2−N2	Sn1−E1 (E=S/Se)	C1‐Sn1‐C4 C2‐Sn2‐C3	Sn1‐C1‐C2 Sn2‐C2‐C1
**3‐Ph**	2.263(2) 2.266(2)	1.368(3) 1.369(3)	1.411(3) 1.417(3)	–	88.0(1) 88.6(1)	128.5(2) 122.0(2)
**3‐DMP**	2.253(2) 2.259(3)	1.376(4) 1.374(4)	1.414(3) 1.417(3)	–	87.4(1) 88.4(1)	124.3(2) 124.9(2)
**4‐Ph**	2.192(5) 2.261(5)	1.379(8) 1.362(8)	1.395(7) 1.404(7)	2.262(1)	94.3(2) 88.7(2)	117.4(4) 128.9(4)
**5‐Ph**	2.195(4) 2.259(4)	1.357(5) 1.365(5)	1.411(5) 1.418(5)	2.388(1)	94.8(1) 88.4(1)	118.0(3) 129.3(3)

We performed DFT calculations at the B3LYP/6‐31G(d) level of theory (LANL2DZ for Sn) for **3‐Ph** to gain further insight into the electronic structures of **3‐Ar**. The NPA (natural population analyses) atomic partial charges (Table S4) calculated using the NBO (natural bond orbital) method indicate that each tin atom in **3‐Ph** (0.94 *e*) carries a positive charge, whereas each of the carbene carbon atoms bears a negative charge of −0.30 *e*. The calculated WBIs (Wiberg Bond Indices) for the Sn−C_ADC_ bonds (0.55) are identical and consistent with the experimental Sn−C_ADC_ bond lengths. The HOMO and HOMO−1 of **3‐Ph** are mainly the s‐type lone‐pair orbitals at the Sn^II^ atoms, whereas the LUMO is located at the imidazole moieties (Figure 3). The HOMO–LUMO energy gap of **3‐Ph** (Δ*E*_H−L_=3.16 eV) is small, which is also manifested by its reactions with chalcogens.

**Figure 3:**
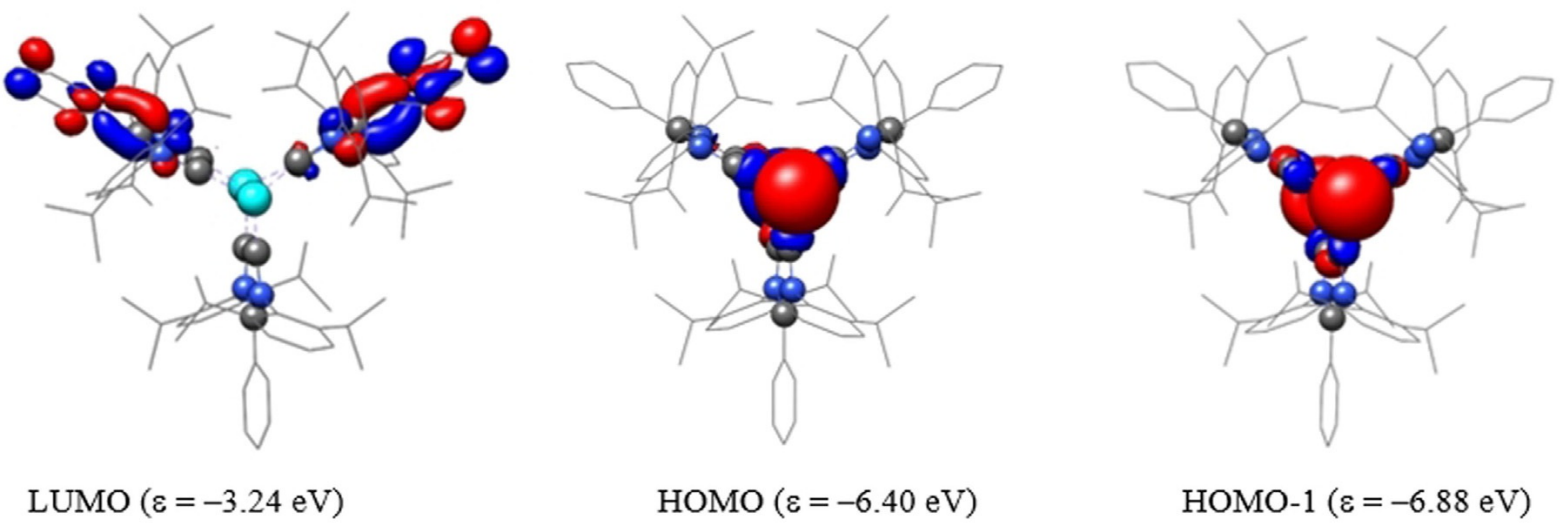
Selected molecular orbitals (isovalue 0.04) of **3‐Ph** calculated at B3LYP/6‐31G(d) level of theory. Hydrogen atoms are omitted for clarity.

Treatment **3‐Ph** and **3‐DMP** each with two equivalents of elemental sulfur led to the formation of mixed valent Sn^II^/Sn^IV^ compounds **4‐Ph** and **4‐DMP**, respectively, as yellow solids in an almost quantitative yield. Similarly, reaction of **3‐Ph** with selenium also gave the mixed valent Sn^II^/Sn^IV^ compound **5‐Ph** (Scheme 2). Both **4‐Ar** and **5‐Ph** feature the dianionic counter anion (SnCl_6_)^2−^, which is assumed to form through the disproportionation of an in situ generated anion (ESnCl_3_)^−^ as follows: 2 (ESnCl_3_)^−^→(SnCl_6_)^2−^+(SnE_2_) or (ESnCl_3_)^−^+(SnCl_3_)^−^→(SnCl_6_)^2−^+(SnE). Calculations show the transformation 2 (SSnCl_3_)^−^→(SnCl_6_)^2−^+(SnS_2_) is thermodynamically favored by Δ*G*=−47.7 kcal mol^−1^. **4‐Ar** and **5‐Ph** do not react further with an excess of chalcogens even at elevated temperature (60–70 °C), indicating that the Sn^II^ moiety of **4‐Ar** and **5‐Ph** is kinetically inert compared to that of **3‐Ar** (see below).

**Scheme 2:**
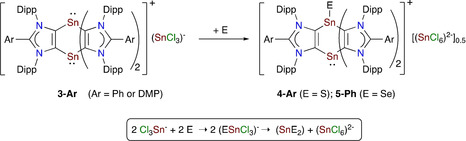
Reactions of distannabarrelenes **3‐Ph** and **3‐DMP** with elemental chalcogens to **4‐Ar** and **5‐Ph**.

The ^1^H NMR spectra of **4‐Ph**, **4‐DMP**, and **5‐Ph** show eight doublets and four septets for the isopropyl groups, which is expected owing to their lower symmetry compared to **3‐Ar**. The ^119^Sn{^1^H} NMR spectrum of **4‐Ph** (−290, −376, and −682 ppm), **4‐DMP** (−342, −366, and −576 ppm), and **5‐Ph** (−290, −376, and −682 ppm) each shows three singlets, which may be assigned to the Sn^II^ and Sn^IV^ nuclei of the cationic part and the Sn^IV^ nucleus of the stannate anion, respectively. The ^119^Sn{^1^H} NMR signals for the SnS moiety of **4‐Ph** (−376) and **4‐DMP** (−366) are high‐field shifted compared to that of Chivers's (−133 ppm)^24^ and Parkin's (−301 ppm)^25^ terminal sulfido compounds with three or four N‐donor substituents at the tin atoms, respectively. The ^77^Se{^1^H} NMR spectrum of **5‐Ph** (−439 ppm) shows a singlet, which is upfield shifted compared to that of Chivers's compound (−174 ppm)^24^ with a four‐coordinated tin atom, suggesting a large polarized nature of the Sn^δ+^−Se^δ−^ bond of **5‐Ph**.15h

Compounds **4‐Ph**, **4‐DMP**, and **5‐Ph** belong to a rare family of thiolate/selenolate derivatives with a terminal Sn‐E bond (Table 1).3h, 26 The solid‐state molecular structures of **4‐Ph** and **5‐Ph** (Figure 4) show the expected bond connectivity. The triclinic unit cell of **4‐Ph** as well as **5‐Ph** contains two cationic fragments, where the counter anion (SnCl_6_)^2−^ resides at the crystallographic center of inversion. The Sn−S bond length of **4‐Ph** (2.262(1) Å) is comparable with that calculated for H_2_Sn=S (2.22 Å)^27^ as well as those of the literature known compounds containing a terminal Sn=S unit (2.25–2.28 Å) with a four coordinated tin atom.^25^, ^28^ This is, however, considerably smaller with respect to known Sn−S single bond lengths (ca. 2.50 Å).^26^, ^29^ The Sn−Se bond length (2.388(5) Å) in **5‐Ph** is in line with the literature known values for Sn=Se double bonds (2.37–2.42 Å) in stannaneselones,^26^, ^30^ but smaller than the Sn−Se single bond length (2.55–2.60 Å).^26^, ^29^ In **4‐Ph** and **5‐Ph**, the Sn^II^−C_ADC_ bond lengths (2.26–2.28 Å) are similar to those of **3‐Ph** (2.24 to 2.27 Å), whereas the Sn^IV^−C_ADC_ bond lengths (2.18–2.20 Å) are slightly shorter than those of **3‐Ph** (Table 1). This bond length trend for Sn^II^ and Sn^IV^ units is expected, which is also in line with the Jurkschat's Sn^IV^ compounds (2.13–2.15 Å) featuring a C4‐bound aNHC.^31^ The C_ADC_‐Sn‐C_ADC_ bond angles of **4‐Ph** at the Sn^II^ center (87.8 to 88.8°) are comparable with those of **3‐Ph** (86.9 to 90.1°), which are, however, larger at the Sn^IV^ center (94.4 to 95.6°).

**Figure 4:**
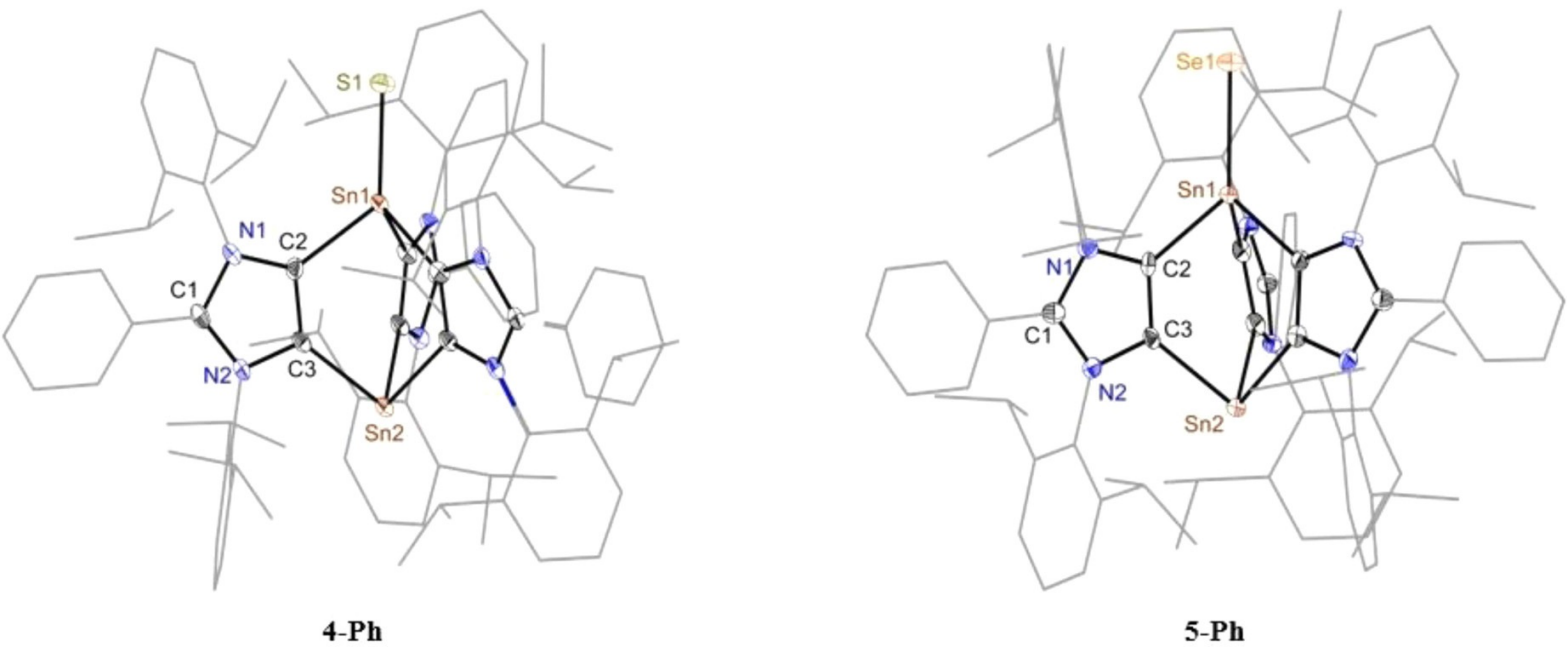
Solid‐state molecular structures of **4‐Ph** and **5‐Ph**. Hydrogen atoms, solvent molecules and the counter anion (SnCl_6_)^2−^ are omitted and aryl groups are shown as wireframe models for clarity.

The computed NPA (at the B3LYP/6‐31G(d) level of theory) show positive charge at the tin atoms of **4‐Ph** (1.79 *e* for Sn^IV^ and 1.55 *e* for Sn^II^) and **5‐Ph** (1.74 for Sn^IV^ and 1.30 *e* for Sn^II^), whereas the sulfur (−0.56 *e* for **4‐Ph**) and selenium (−0.27 *e* for **5‐Ph**) atoms have a negative charge. This is consistent with the electronegativity difference between Sn and S/Se atoms and suggests that the Sn=E (E=S, Se) bond in **4‐Ph** and **5‐Ph** is polarized towards the chalcogen atom. As expected, the carbene carbon atoms in **4‐Ph** and **5‐Ph** also bear negative charges.^17^ The NBO analyses also confirm that the Sn=E bond of **4‐Ph** and **5‐Ph** is polarized as evidenced by the WBI (Wiberg Bond Indices) of 1.08 and 0.96, respectively. Similar to compound **3‐Ph**, the HOMO of **4‐Ph** (−12.05 eV) and **5‐Ph** (−11.79 eV) each is mainly the lone pair orbital at the Sn^II^ moiety with a small contribution from the *p*‐orbital of sulfur or selenium atom (Figure 5). The HOMO of **4‐Ph** and **5‐Ph** is, however, considerably stabilized compared to that of **3‐Ph** (−6.40 eV), rationalizing their kinetic inertness towards further oxidation with chalcogens. The LUMO of **4‐Ph** and **5‐Ph** also show contribution from s‐orbital of Sn^II^ and *p*‐orbital of sulfur and selenium (see the Supporting Information).

**Figure 5:**
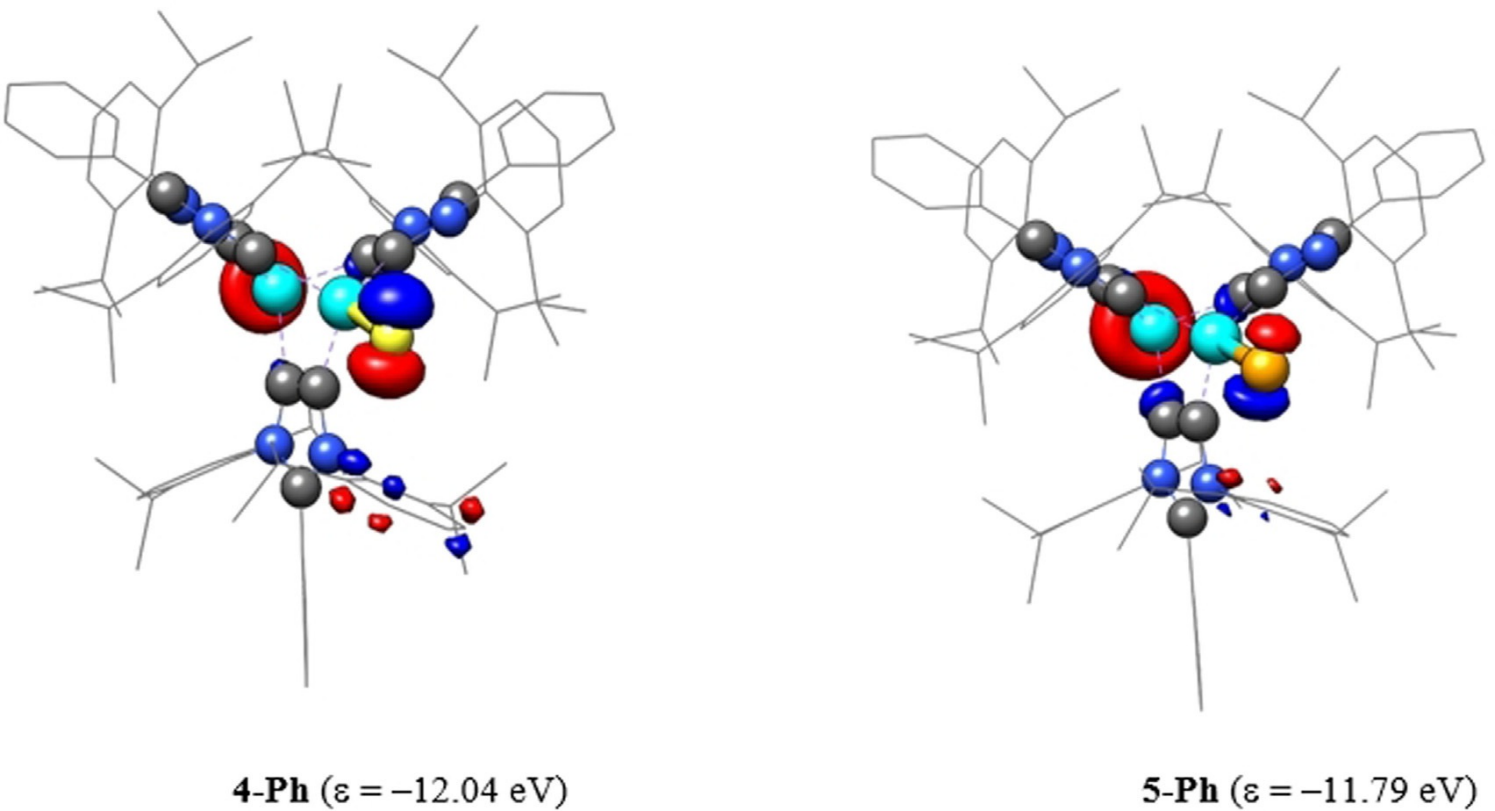
HOMOs (isovalue 0.04) of **4‐Ph** and **5‐Ph** calculated at B3LYP/6‐31G(d) level of theory. Hydrogen atoms are omitted for clarity.

In conclusion, the first distannabarrelenes **3‐Ar** featuring two all carbon substituted stannylenes have been reported as crystalline solids. These ionic compounds are derived from ADCs and feature cationic bicyclo[2.2.2]‐1,4‐bis‐stannylene and anionic SnCl_3_ units. **3‐Ar** selectively react with chalcogens (E=S or Se) to form mixed‐valence Sn^II^/Sn^IV^ compounds **4‐Ar** and **5‐Ph**, in which the barrelene moiety remains intact. The anionic part (SnCl_3_) of **3‐Ar** also reacts with chalcogens to form the (SnCl_6_)^2−^ anion by the disproportionation of putative (ESnCl_3_)^−^ species. This report emphasizes the suitability of ADCs for accessing heterocyclic compounds featuring low‐valent main‐group elements with interesting bonding motifs, which may lead to new discoveries in synthesis and materials science.

## Conflict of interest

The authors declare no conflict of interest.

## Supporting information

As a service to our authors and readers, this journal provides supporting information supplied by the authors. Such materials are peer reviewed and may be re‐organized for online delivery, but are not copy‐edited or typeset. Technical support issues arising from supporting information (other than missing files) should be addressed to the authors.

Supplementary
