# Supplementary material for: Distannabarrelenes with Three Coordinated SnII Atoms
Source: Chemistry. 2020 Jul 28;26(49):11113–8. doi: 10.1002/chem.202001432 (PMC7496072; doi:10.1002/chem.202001432)
Supplement: Supplementary file 1 — Supplementary [file CHEM-26-11113-s001.pdf]

Chemistry–A European Journal

Supporting Information

Distannabarrelenes with Three Coordinated Sn^{II} Atoms

Mahendra K. Sharma, Timo Glodde, Beate Neumann, Hans-Georg Stammler, and Rajendra S. Ghadwal*[a]

Contents

Experimental Section 1

Plots of the NMR Spectra 4

Crystallographic Details 12

Computational Details 12

References 46

Experimental Section

All experiments and manipulations were carried out under an inert gas (Ar or N_2) atmosphere using standard *Schlenk* techniques or an MBraun LABmaster Pro glovebox. Solvents (THF, benzene, *n*-hexane over NaK; dichloromethane (DCM) over CaH_2) were dried by refluxing over appropriate drying agents, distilled prior to use, and stored over 3Å molecular sieve. (IPr^{Ph})Cl (**1-Ph**) and (IPr^{DMP})Cl (**1-DMP**) were prepared by the anion-exchange of the corresponding bromide salts.[1] $SnCl_2$ (Acros), sulphur, and selenium (Sigma) powders were used as supplied. NMR spectra were recorded using a Bruker Avance III 500HD NMR spectrometer. Chemical shifts are given in δ ppm and referenced to the solvent residual peak(s).[2] Melting points were measured using a Büchi B-545 melting point apparatus.

Synthesis of [Sn_2(ADC^{Ph})$_3$]$SnCl_3$ (3-Ph): To a *Schlenk* flask containing a THF suspension of (IPr^{Ph})Cl (**1-Ph**) (2.0 g, 3.99 mmol) was added *n*-BuLi (2.5 M, 3.27 mL, 8.18 mmol) at −60 °C. The resulting reaction mixture was slowly brought up to room temperature and stirred for 30 minutes to get a clear light brown solution of ADC^{Ph} (**2-Ph**). It was cooled to −40 °C and a THF solution of $SnCl_2$ (0.76 g, 4.0 mmol) was added. The resulting solution was stirred at room temperature for 12 h and then the volatiles were removed in vacuo. The yellow residue was extracted with DCM (20 mL) and filtered through a plug of Celite. Removal of DCM from the filtrate in vacuo gave a yellow solid, which was washed with benzene (10 mL) and dried. Yield: 76%, 1.88 g. M.p. 198 °C (dec.). X-ray quality single crystals were grown by a slow diffusion of *n*-hexane into a saturated DCM solution of **3-Ph**. Elemental analysis (%), calcd for $C_{99}H_{117}Cl_3N_6Sn_3$ (1854.55) **3-Ph**: C, 64.15; H, 6.36; N, 4.53; found: C, 64.67; H, 6.97; N, 4.77. ^{1}H NMR (500 MHz, CD_2Cl_2, 298 K): δ = 7.45 (t, J = 7.8 Hz, 2H, *p*-C_6H_3), 7.35 (d, J = 7.4 Hz, 2H, *m*-C_6H_3), 7.13 (t, J = 7.4 Hz, 1H, *p*-C_6H_5), 7.09 (d, J = 7.7 Hz, 2H, *m*-C_6H_3), 6.96 (t, J = 7.8 Hz, 2H, C_6H_5), 6.87 (d, J = 8.2 Hz, 2H, C_6H_5), 2.92-2.98 (m, 2H, C*H*(CH_3)$_2$), 1.74-1.79 (m, 2H, C*H*(CH_3)$_2$), 1.11 (d, J = 6.6 Hz, 6H, CH(C*H*$_3$)$_2$), 0.93 (d, J = 6.7 Hz, 6H, CH(C*H*$_3$)$_2$), 0.81 (d, J = 6.6 Hz, 6H, CH(C*H*$_3$)$_2$), 0.10 (d, J = 6.7 Hz, 6H, CH(C*H*$_3$)$_2$) ppm. ^{13}C{^{1}H} NMR (125 MHz, THF-d_8, 298 K): δ = 171.5, 148.2, 145.5, 145.0, 136.4, 131.4, 130.8 (*C*$_6$H$_3$); 129.1, 129.0, 126.8, 126.4, 124.7 (*C*$_6$H$_5$); 29.6, 29.0 (CH(*C*H$_3$)$_2$; 27.4, 24.3, 22.8 (*C*H$_3$). ^{119}Sn{^{1}H} NMR (186 MHz, THF-d_8, 298 K): δ = −298.6 ppm.

Synthesis of [Sn_2(ADC^{DMP})$_3$]$SnCl_3$ (3-DMP): Compound **3-DMP** was synthesized as an off-white solid by adopting a similar protocol as described for **3-Ph** using (IPr^{DMP})Cl (**1-DMP**) (5.0 g, 9.19 mmol), *n*-BuLi (2.5 M, 7.53 mL, 18.83 mmol) and $SnCl_2$ (1.74 g, 9.19 mmol). Yield: 95%, 5.77 g. Mp: 189 °C (dec.). Elemental analysis (%), calcd for $C_{105}H_{132}Cl_3N_9Sn_3$ (1983.67) **3-DMP**: C, 63.61; H, 6.71; N, 6.36; found: C, 63.97; H, 7.11; N, 6.57. ^{1}H NMR (500 MHz, CD_2Cl_2, 298 K): δ = 7.42 (t, J = 7.8 Hz, 2H, *p*-C_6H_3), 7.31 (d, J = 7.9 Hz, 2H, *m*-C_6H_3), 7.11 (d, J = 8.9 Hz, 2H, *m*-C_6H_3), 6.63 (d, J = 9.3 Hz, 2H, C_6H_4), 6.14 (d, J = 9.3 Hz, 2H, C_6H_4), 2.95-3.00 (m, 2H, C*H*(CH_3)$_2$), 2.78 (s, 6H,

$N(CH_3)_2$), 1.80-1.85 (m, 2H, $CH(CH_3)_2$), 1.08 (d, J = 6.6 Hz, 6H, $CH(CH_3)_2$), 0.95 (d, J = 6.7 Hz, 6H, $CH(CH_3)_2$), 0.80 (d, J = 6.7 Hz, 6H, $CH(CH_3)_2$), 0.21 (d, J = 6.8 Hz, 6H, $CH(CH_3)_2$) ppm. $^{13}C\{^1H\}$ NMR (125 MHz, CD_2Cl_2, 298 K): δ = 169.4, 151.0, 148.4, 145.8, 144.9, 136.6, 131.4, 130.3 (C_6H_3); 128.9, 125.9, 125.6, 111.1, 110.7 (C_6H_5); 40.0 ($N(CH_3)_2$); 29.1, 28.6 ($CH(CH_3)_2$; 27.0, 25.5, 23.8, 22.8 (CH_3). $^{119}Sn\{^1H\}$ NMR (186 MHz, CD_2Cl_2, 298 K): δ = −297.5 ppm.

Synthesis of $[Sn_2(ADC^{Ph})_3(S)]_2SnCl_6$ (4-Ph): To a *Schlenk* flask containing **3-Ph** (0.5 g, 0.27 mmol) and sulphur powder (31 mg, 0.81 mmol) was added 20 mL DCM at room temperature. The resulting reaction mixture was stirred overnight. Removal of the volatiles in vacuo gave a yellow residue, which was washed with 10 mL *n*-hexane and dried. Yield: 0.48 g, 98%. M.p. 210 °C (dec.). X-ray quality single crystals were grown by storing a saturated THF solution of **4-Ph** at –40 °C for 1 day. Elem. Anal. calcd. for $C_{99}H_{117}Cl_3N_6SSn_{2.5}$ (1826.57) **4-Ph**: C, 63.06; H, 6.25; N, 4.46, found: C, 63.39; H, 6.51; N, 4.78. ^1H NMR (500 MHz, CD_2Cl_2, 298 K): δ = 7.55 (t, J = 7.5 Hz, 1H, p-C_6H_3), 7.48 (t, J = 7.5 Hz, 1H, p-C_6H_3), 7.40 (m, 2H, m-C_6H_3), 7.18 (m, 2H, m-C_6H_3), 7.10 (d, J = 7.8 Hz, 1H, p-C_6H_5), 6.97 (t, J = 7.5 Hz, 2H, C_6H_5), 6.91 (d, J = 8.0 Hz, 2H, C_6H_5), 3.01-3.06 (m, 1H, $CH(CH_3)_2$), 2.77-2.82 (m, 1H, $CH(CH_3)_2$), 1.82 (br, 1H, $CH(CH_3)_2$), 1.65-1.70 (m, 1H, $CH(CH_3)_2$), 1.28 (d, J = 6.2 Hz, 3H, $CH(CH_3)_2$), 1.12 (d, J = 6.1 Hz, 3H, $CH(CH_3)_2$), 1.07 (d, J = 6.3 Hz, 3H, $CH(CH_3)_2$), 1.00 (d, J = 6.4 Hz, 3H, $CH(CH_3)_2$), 0.96 (d, J = 6.3 Hz, 3H, $CH(CH_3)_2$), 0.75 (d, J = 6.2 Hz, 3H, $CH(CH_3)_2$), 0.15 (d, J = 6.4 Hz, 3H, $CH(CH_3)_2$), 0.07 (d, J = 6.4 Hz, 3H, $CH(CH_3)_2$) ppm. $^{13}C\{^1H\}$ NMR (125 MHz, CD_2Cl_2, 298 K): δ = 166.2, 153.0, 149.0, 145.8, 145.2, 144.5, 143.8, 135.3, 133.8, 131.7, 131.3, 130.7 (C_6H_3); 128.8, 128.6, 126.8, 126.6, 126.1, 125.6, 123.1 (C_6H_5); 29.9, 29.6, 28.8, 28.7 ($CH(CH_3)_2$; 27.1, 26.8, 25.7, 25.5, 24.0, 23.8, 22.7, 22.4 (CH_3). $^{119}Sn\{^1H\}$ NMR (186 MHz, CD_2Cl_2, 298 K): δ = −289.9, −375.9, −682.0 ppm.

Synthesis of $[Sn_2(ADC^{DMP})_3(S)]_2SnCl_6$ (4-DMP): Compound **4-DMP** was synthesized as an off white solid by adopting a similar protocol as described for **4-Ph** using **3-DMP** (0.5 g, 0.25 mmol) and sulphur powder (30 mg, 0.76 mmol). Yield: 99%, 0.49 g. Mp: 197 °C (dec.). Elem. Anal. calcd. for $C_{105}H_{132}Cl_3N_9SSn_{2.5}$ (1955.70) **4-DMP**: C, 62.59; H, 6.60; N, 6.26, found: C, 63.07; H, 6.97; N, 6.49. ^1H NMR (500 MHz, CD_2Cl_2, 298 K): δ = 7.50 (t, J = 7.5 Hz, 1H, p-C_6H_3), 7.45 (t, J = 7.6 Hz, 1H, p-C_6H_3), 7.35 (d, J = 6.0 Hz, 2H, m-C_6H_3), 7.19 (d, J = 7.2 Hz, 1H, m-C_6H_3), 7.12 (d, J = 7.3 Hz, 1H, m-C_6H_3), 6.63 (d, J = 8.8Hz, 2H, C_6H_4), 6.11 (d, J = 8.8 Hz, 2H, C_6H_4), 3.03-3.06 (m, 1H, $CH(CH_3)_2$), 2.79 (br, 7H, $CH(CH_3)_2$, $N(CH_3)_2$), 1.85-1.88 (m, 1H, $CH(CH_3)_2$), 1.74-1.78 (m, 1H, $CH(CH_3)_2$), 1.27 (d, J = 6.0 Hz, 3H, $CH(CH_3)_2$), 1.08 (d, J = 6.0 Hz, 3H, $CH(CH_3)_2$), 1.03 (d, J = 6.0 Hz, 6H, $CH(CH_3)_2$), 0.98 (d, J = 6.3 Hz, 3H, $CH(CH_3)_2$), 0.73 (d, J = 6.3 Hz, 3H, $CH(CH_3)_2$), 0.26 (d, J = 6.3 Hz, 3H, $CH(CH_3)_2$), 0.19 (d, J = 6.3 Hz, 3H, $CH(CH_3)_2$) ppm. $^{13}C\{^1H\}$ NMR (125 MHz, CD_2Cl_2, 298 K): δ = 164.6, 151.7, 151.4, 149.9, 149.7, 146.1, 145.7, 145.5, 144.9, 144.3, 144.0, 136.1, 134.7, 132.7, 131.9, 131.3, 131.1, 130.7 (C_6H_3); 128.8, 126.4, 126.2, 125.7, 125.4, 110.4, 110.2, 109.4, 108.1

(C_6H_4); 39.9 ($N(CH_3)_2$); 30.4, 29.9, 29.5, 28.8, 28.6 ($CH(CH_3)_2$; 27.0, 26.6, 25.6, 25.4, 23.8, 23.5, 23.3, 23.1, 22.9, 22.7 (CH_3). $^{119}Sn\{^1H\}$ NMR (186 MHz, CD_2Cl_2, 298 K): δ = −342.6, −366.2, −576.2 ppm.

Synthesis of $[Sn_2(ADC^{Ph})_3(Se)]_2SnCl_6$ (5-Ph): To a *Schlenk* flask containing **3-Ph** (0.5 g, 0.27 mmol) and selenium powder (100 mg, 1.35 mmol) was added 20 mL THF at room temperature. The resulting reaction mixture was heated at 60 °C overnight. Then, the excess selenium was removed by filtration and the filtrate was dried in vacuo to give an orange residue, which was washed with 10 mL benzene and dried. Yield: 0.5 g, 99%. M.p. 208 °C (dec.). X-ray quality single crystals were grown by storing a saturated THF solution of **5-Ph** at –40 °C for 1 day. Elem. Anal. calcd. for $C_{99}H_{117}Cl_3N_6SeSn_{2.5}$ (1874.51) **5-Ph**: C, 61.53; H, 6.10; N, 4.35, found: C, 61.97; H, 6.65; N, 4.57. ^1H NMR (500 MHz, CD_2Cl_2, 298 K): δ = 7.55 (t, J = 7.8 Hz, 1H, *p*-C_6H_3), 7.50 (t, J = 7.8 Hz, 1H, *p*-C_6H_3), 7.40 (m, 2H, *m*-C_6H_3), 7.18 (t, J = 8.6 Hz, 2H, *m*-C_6H_3), 7.11 (d, J = 7.7 Hz, 1H, *p*-C_6H_5), 6.96 (t, J = 7.9 Hz, 2H, *m*-C_6H_5), 6.90 (d, J = 8.3 Hz, 2H, *o*-C_6H_5), 3.05-3.11 (m, 1H, $CH(CH_3)_2$), 2.75-2.82 (m, 1H, $CH(CH_3)_2$), 1.81-1.85 (m, 1H, $CH(CH_3)_2$), 1.63-1.68 (m, 1H, $CH(CH_3)_2$), 1.33 (d, J = 6.5 Hz, 3H, $CH(CH_3)_2$), 1.12 (d, J = 6.5 Hz, 3H, $CH(CH_3)_2$), 1.07 (d, J = 6.6 Hz, 3H, $CH(CH_3)_2$), 1.01 (d, J = 6.7 Hz, 3H, $CH(CH_3)_2$), 0.97 (d, J = 6.6 Hz, 3H, $CH(CH_3)_2$), 0.74 (d, J = 6.6 Hz, 3H, $CH(CH_3)_2$), 0.16 (d, J = 6.7 Hz, 3H, $CH(CH_3)_2$), 0.07 (d, J = 7.3 Hz, 3H, $CH(CH_3)_2$) ppm. $^{13}C\{^1H\}$ NMR (125 MHz, CD_2Cl_2, 298 K): δ = 166.3, 150.6, 149.0, 145.7, 145.3, 144.6, 144.0, 135.4, 133.8, 131.7, 131.4, 130.7 (C_6H_3); 128.6, 126.8, 126.7, 126.1, 125.7, 123.1 (C_6H_5); 30.0, 29.6, 28.8, 28.6 ($CH(CH_3)_2$; 27.1, 26.8, 26.1, 25.7, 25.4, 24.4, 23.8, 22.7, 22.3 (CH_3). $^{119}Sn\{^1H\}$ NMR (186 MHz, CD_2Cl_2, 298 K): δ = –289.9, –375.9, –681.8 ppm. $^{77}Se(^1H)$ NMR (95 MHz, CD_2Cl_2, 298 K): δ = –439.1 ppm.

Plots of the NMR Spectra

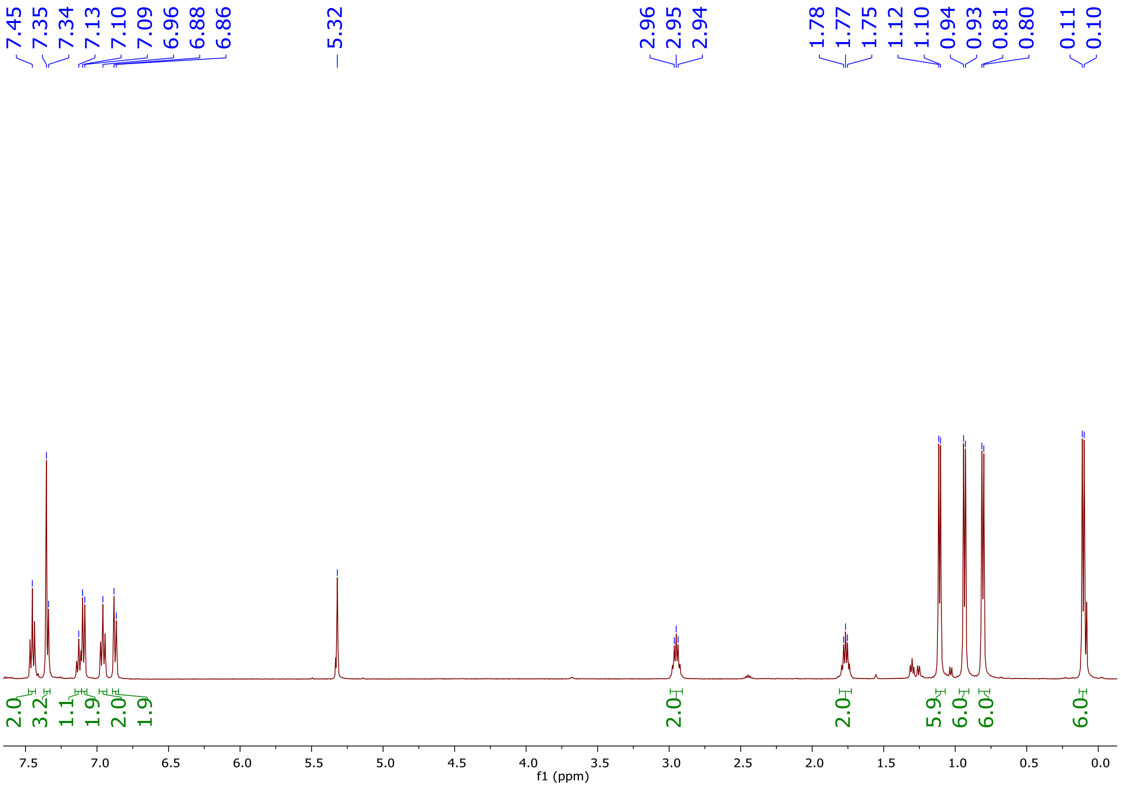

Figure S1. ^{1}H NMR (500 MHz, CD_2Cl_2, 298 K,) spectrum of compound **3-Ph**.

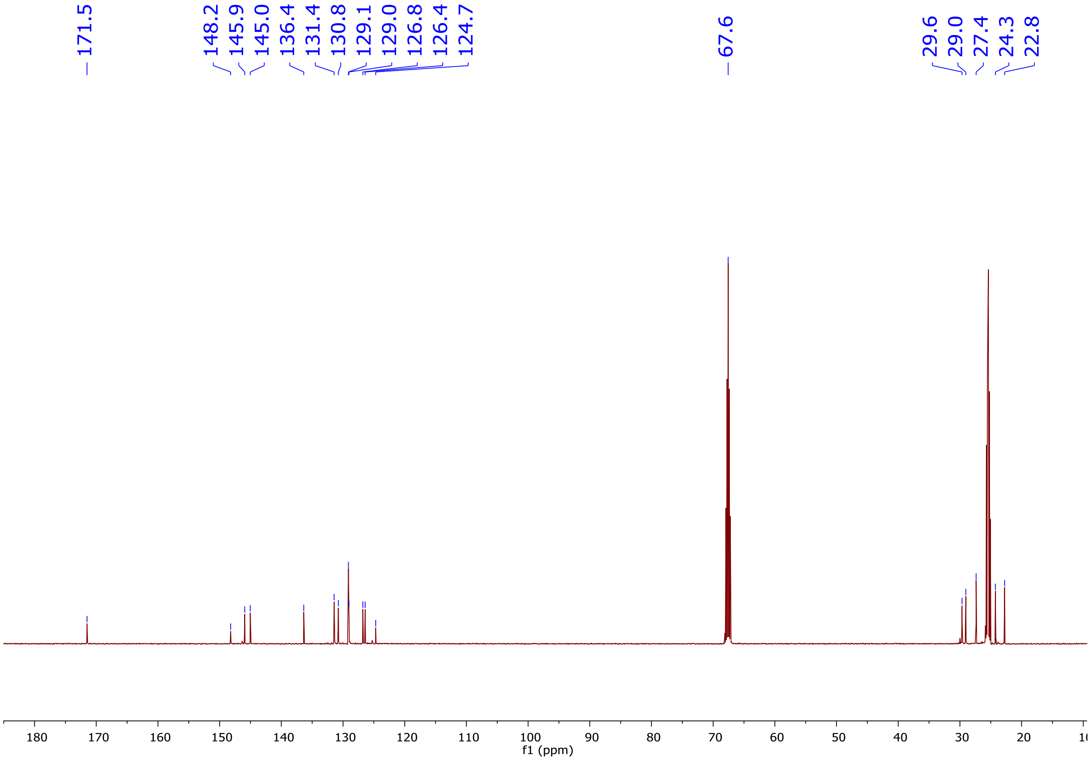

Figure S2 . ^{13}C{^{1}H} NMR (125 MHz, THF-d_8, 298 K) spectrum of compound **3-Ph**.

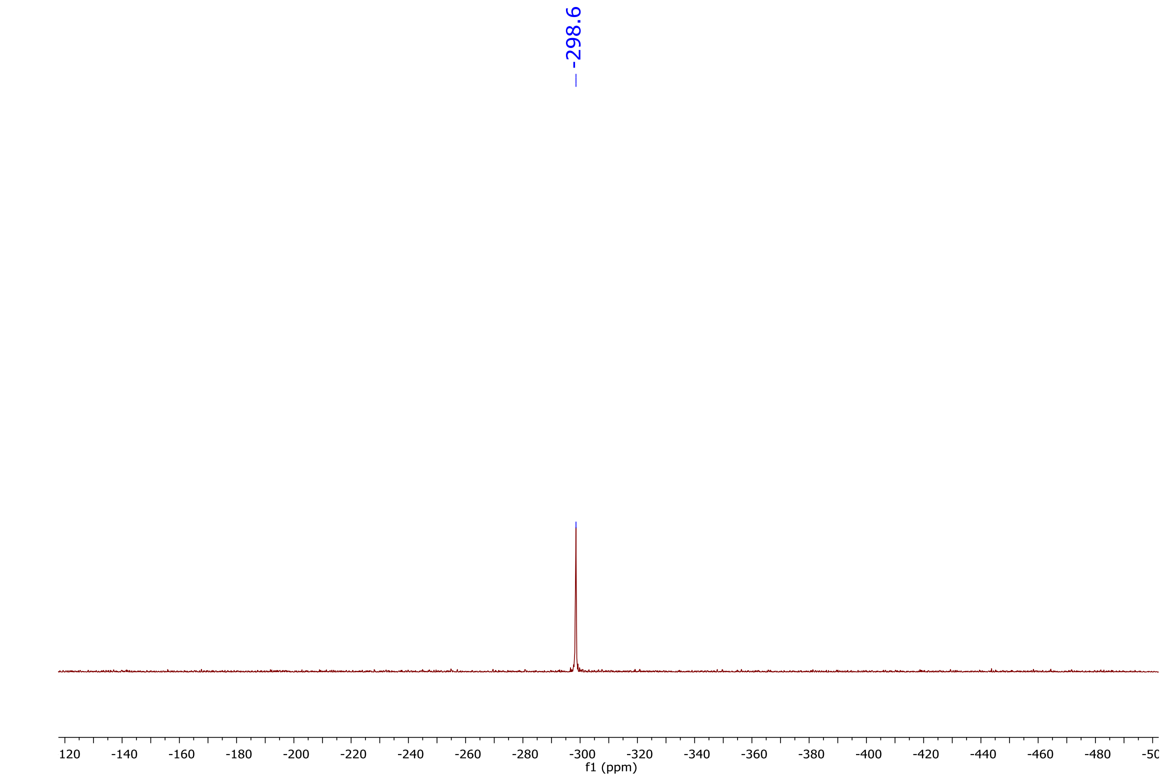

Figure S3. $^{119}Sn\{^{1}H\}$ NMR (186 MHz, THF-d_8, 298 K) spectrum of compound **3-Ph**.

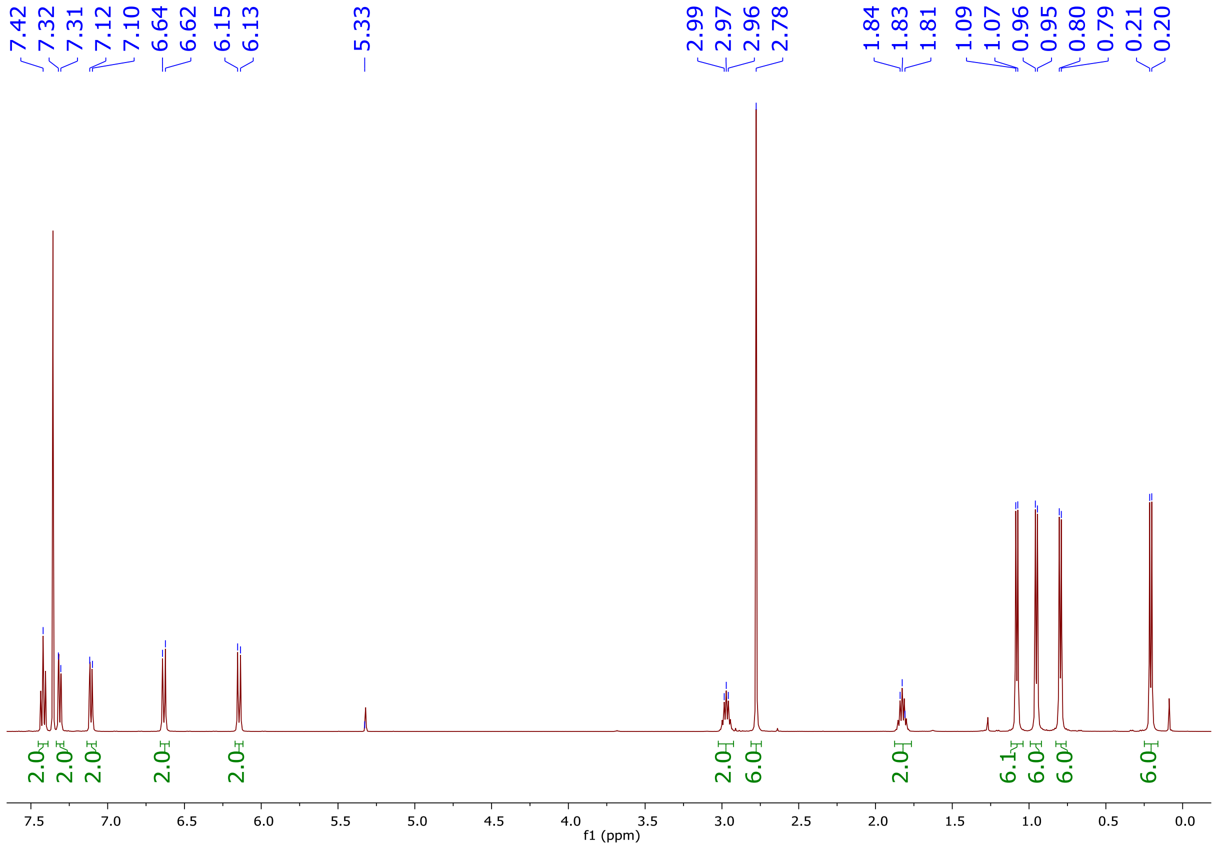

Figure S4. ^{1}H NMR (500 MHz, CD_2Cl_2, 298 K) spectrum of compound **3-DMP**.

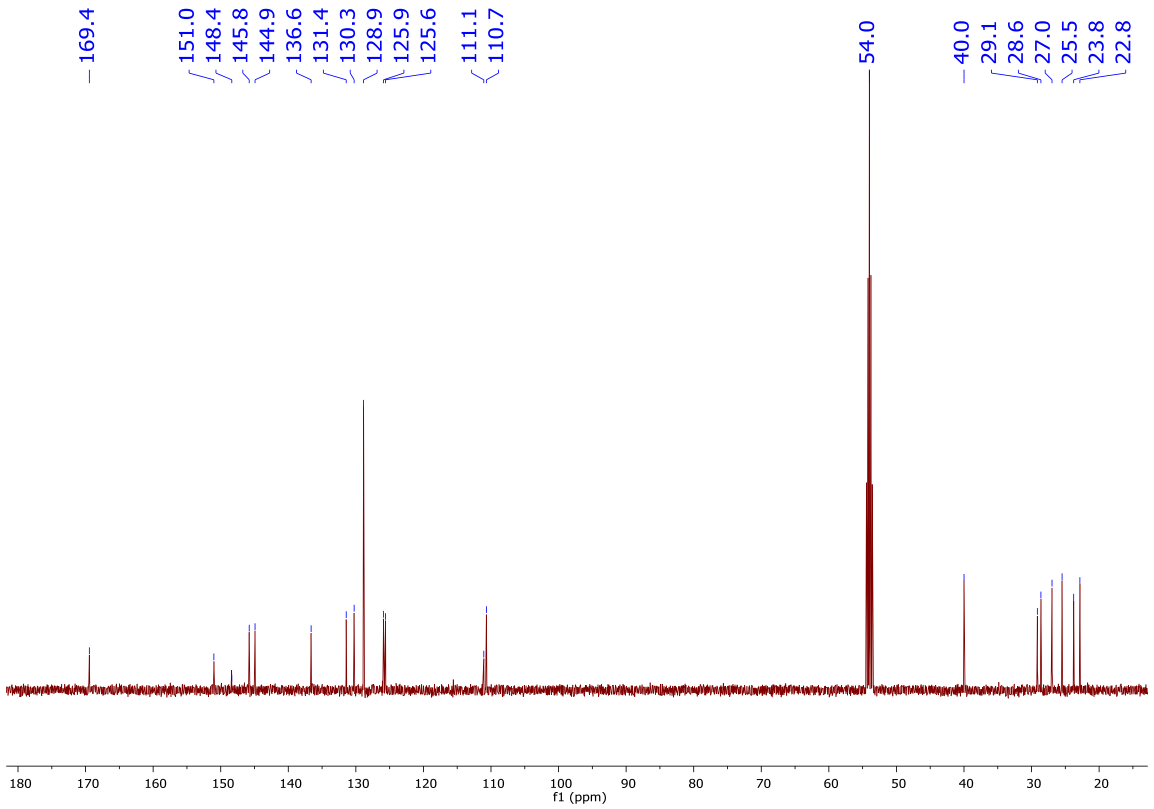

Figure S5. $^{13}C\{^{1}H\}$ NMR (125 MHz, CD_2Cl_2, 298 K,) spectrum of compound **3-DMP**.

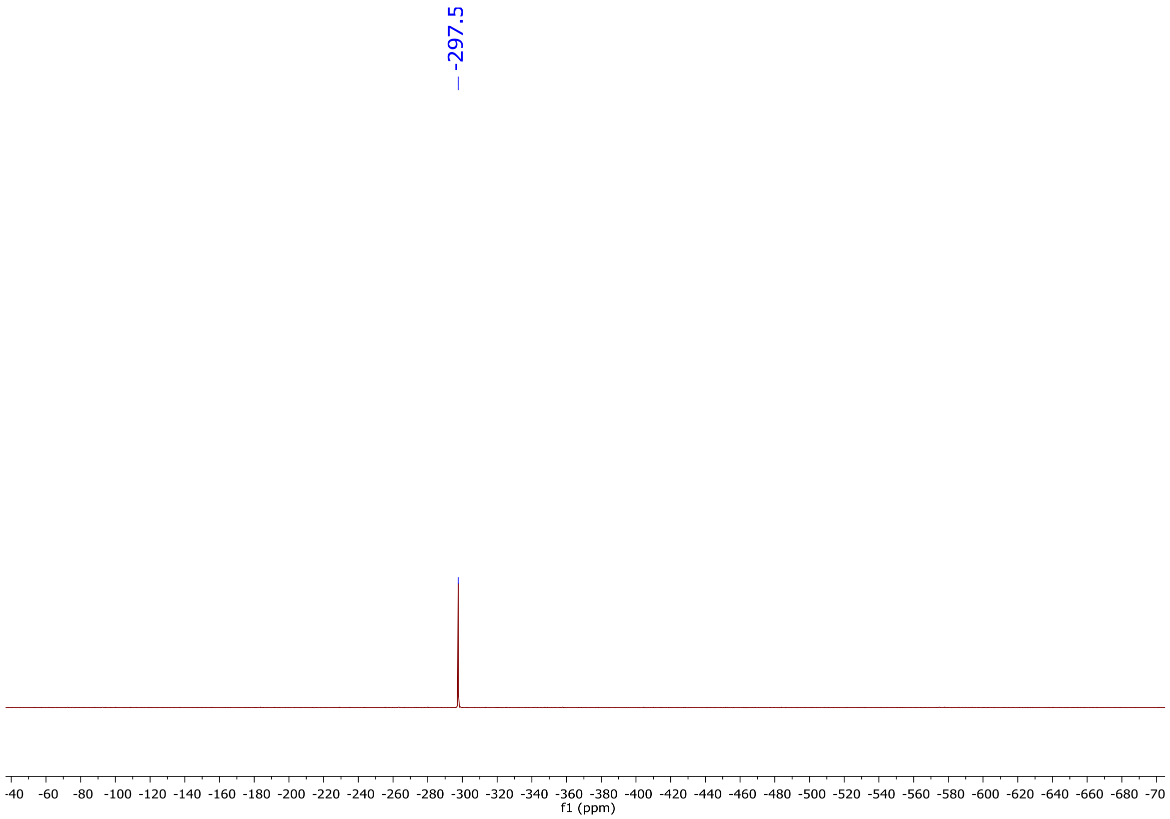

Figure S6. ^{119}Sn{^{1}H} NMR (186 MHz, CD_2Cl_2, 298 K) spectrum of compound **3-DMP**.

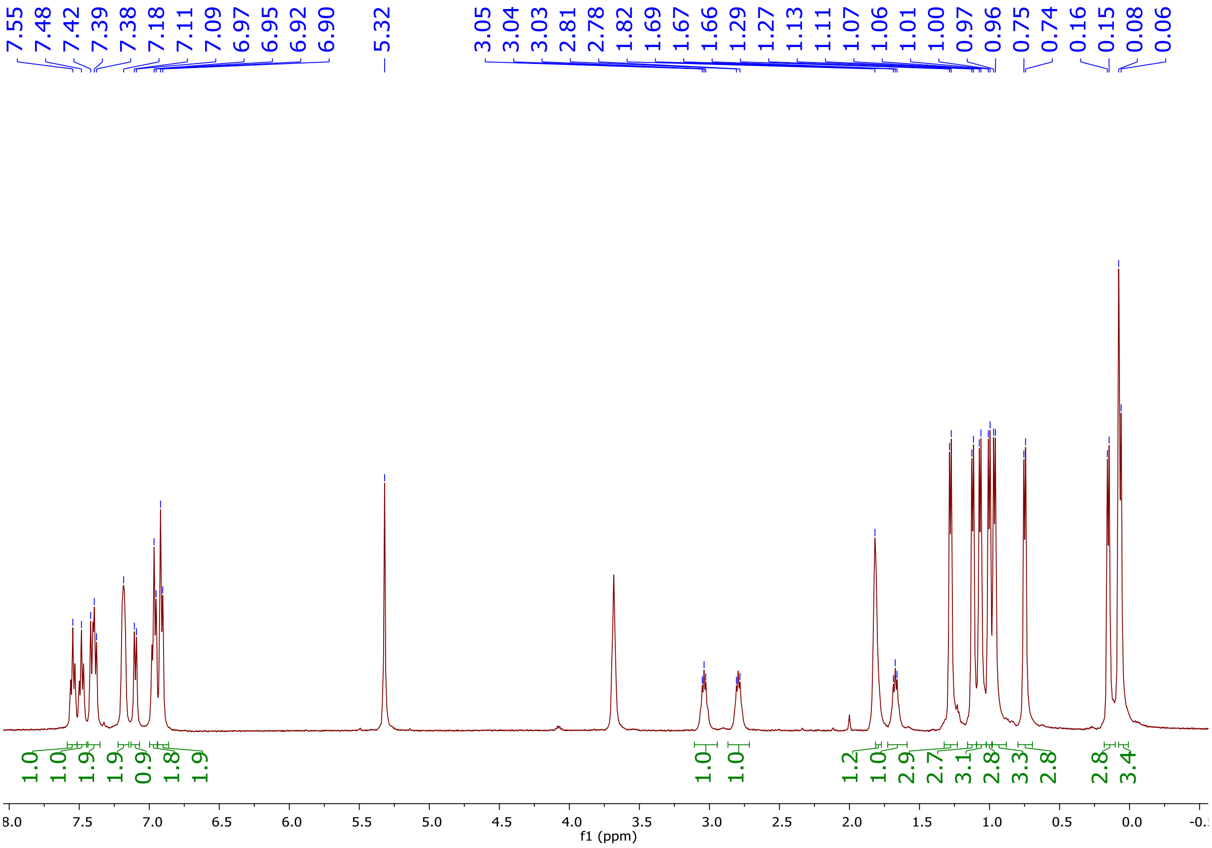

Figure S7. ^{1}H NMR (500 MHz, CD_2Cl_2, 298 K) spectrum of compound **4-Ph**.

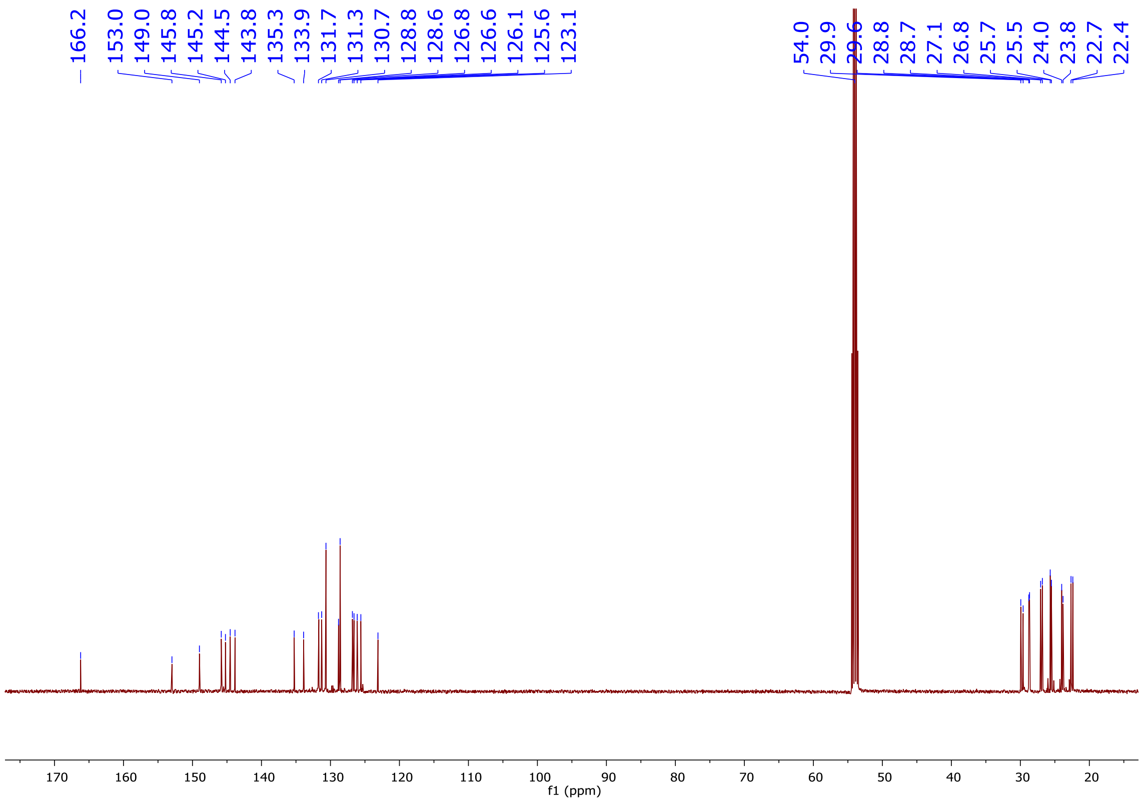

Figure S8. $^{13}C\{^{1}H\}$ NMR (125 MHz, CD_2Cl_2, 298 K) spectrum of compound **4-Ph**.

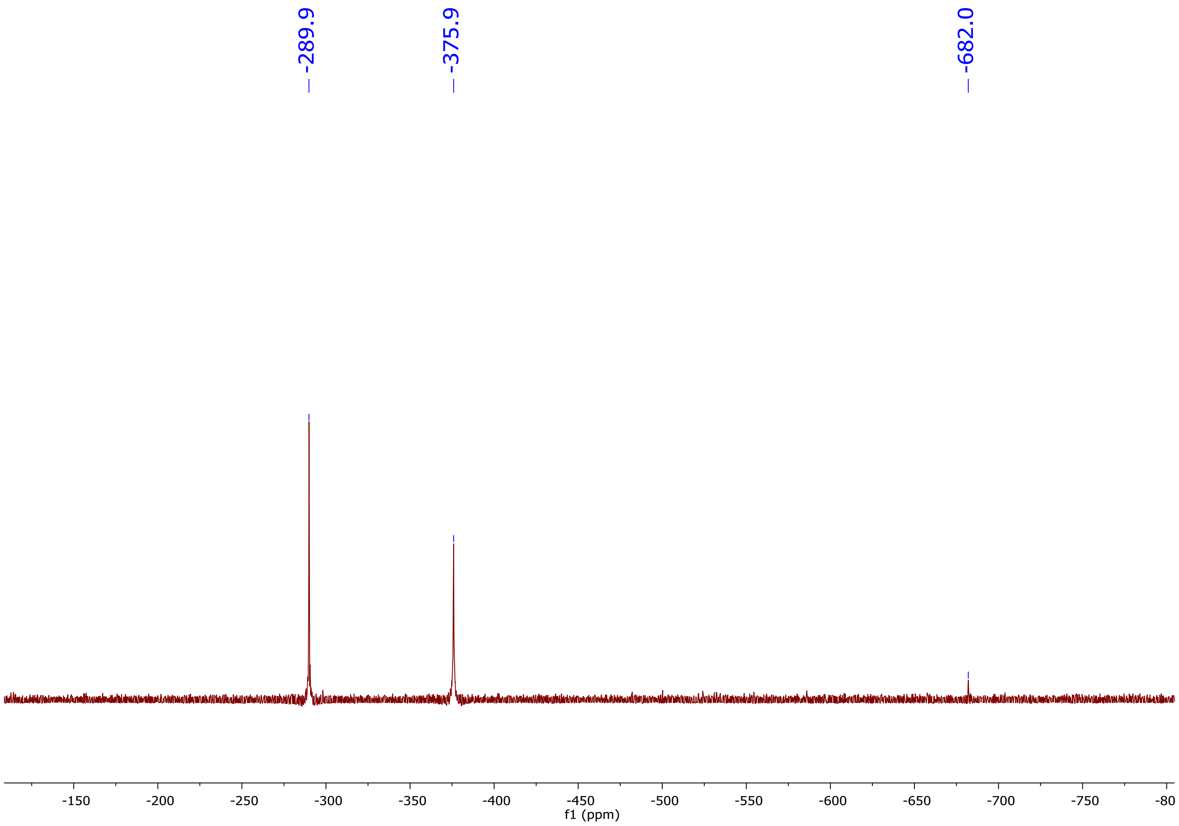

Figure S9. $^{119}Sn\{^{1}H\}$ NMR (186 MHz, CD_2Cl_2, 298 K) spectrum of compound **4-Ph**.

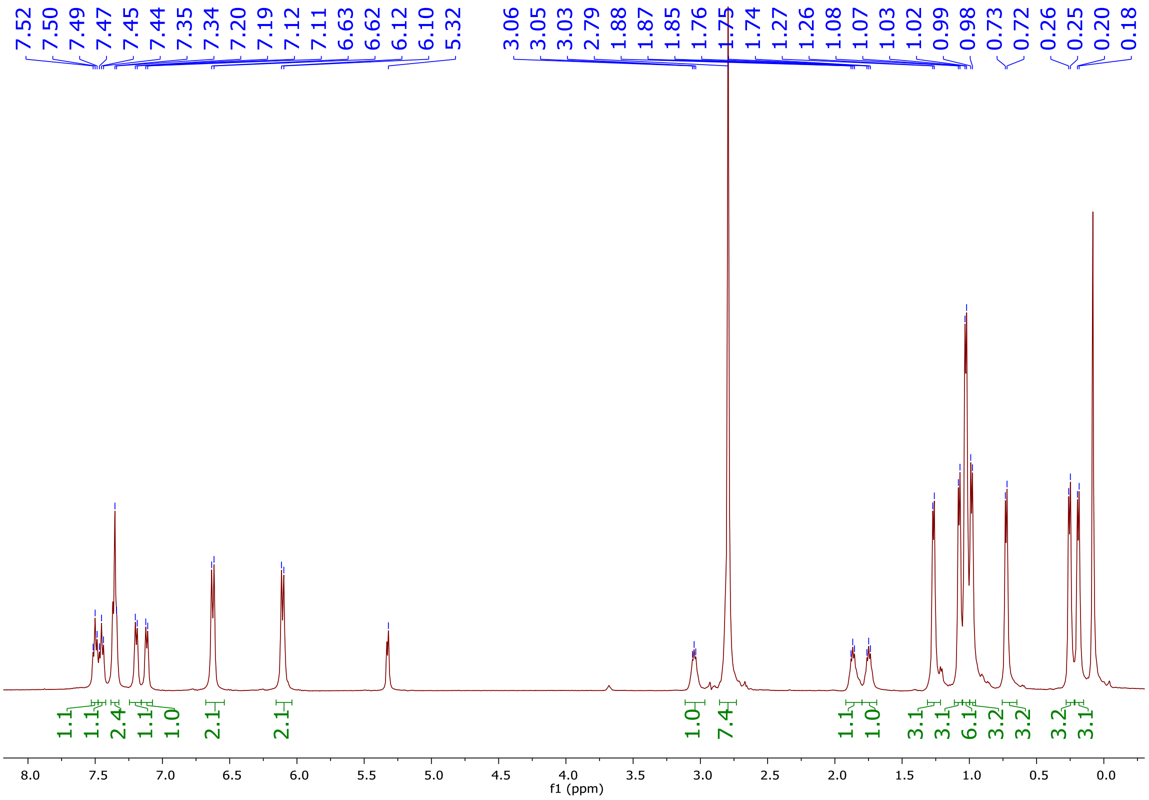

Figure S10. ^{1}H NMR (500 MHz, CD_2Cl_2, 298 K) spectrum of compound **4-DMP**.

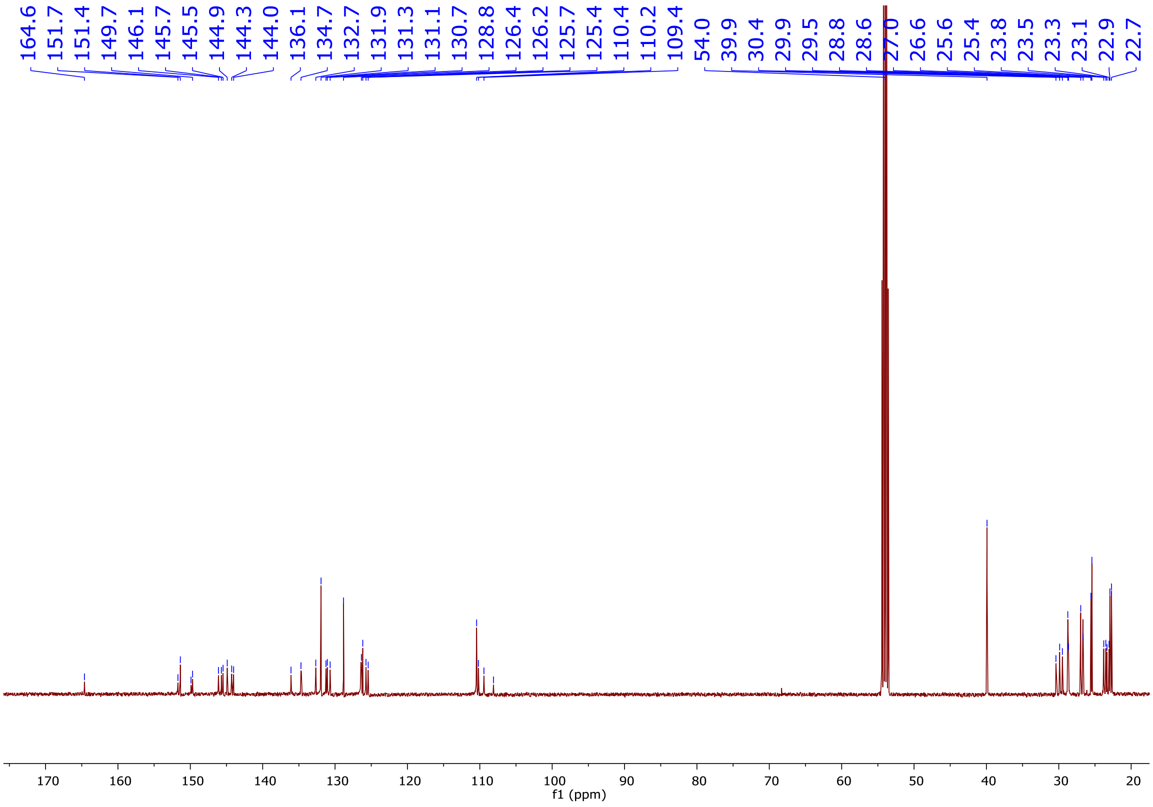

Figure S11. $^{13}C\{^{1}H\}$ NMR (125 MHz, CD_2Cl_2, 298 K) spectrum of compound **4-DMP**.

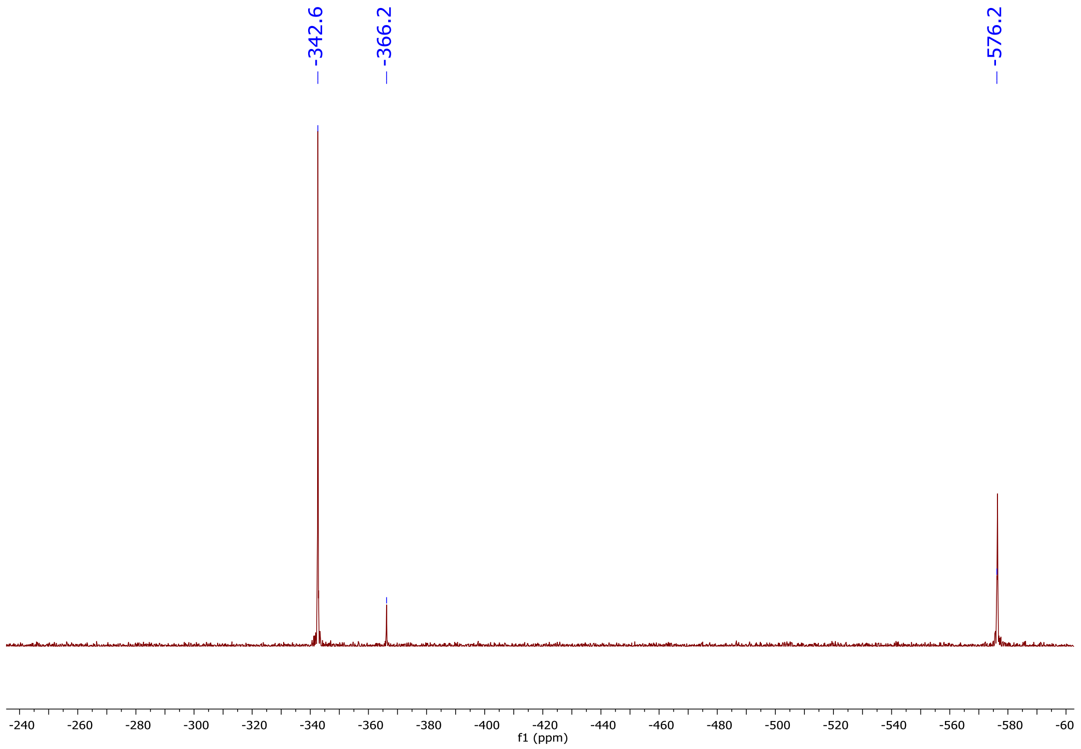

Figure S12. $^{119}Sn\{^{1}H\}$ NMR (186 MHz, CD_2Cl_2, 298 K) spectrum of compound **4-DMP**.

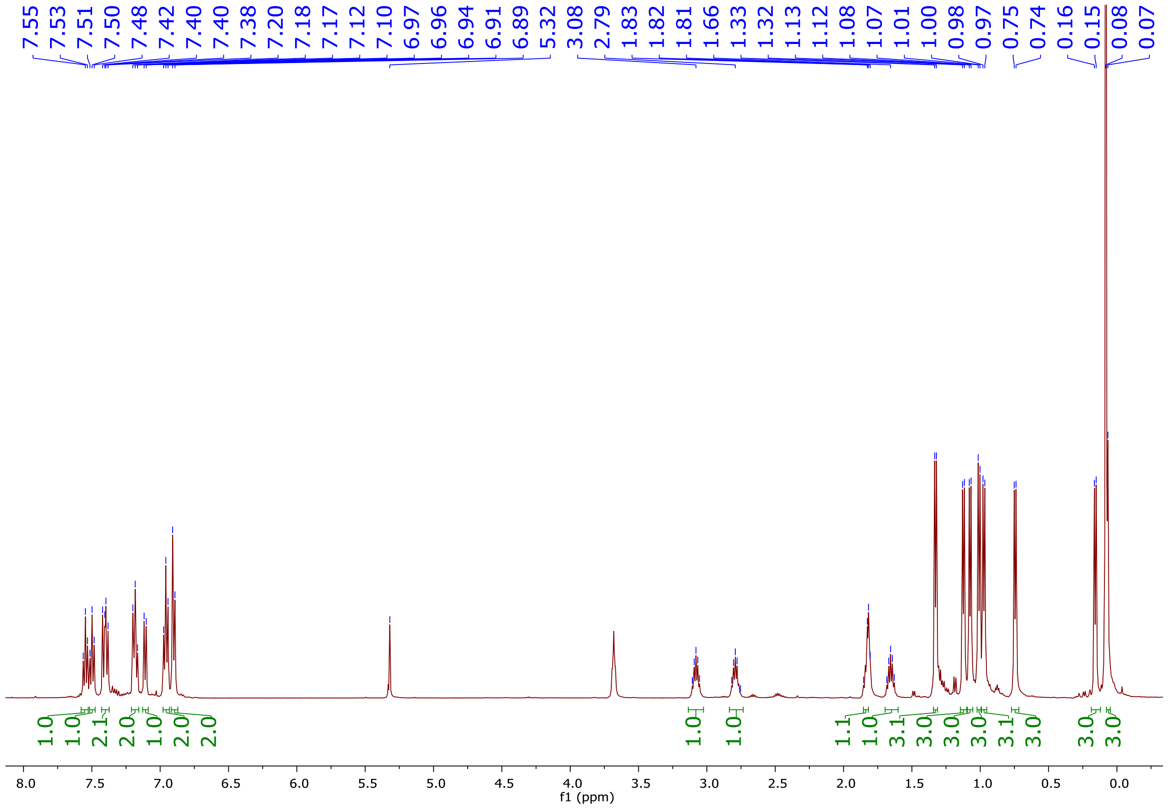

Figure S13. ^{1}H NMR (500 MHz, CD_2Cl_2, 298 K) spectrum of compound **5-Ph**.

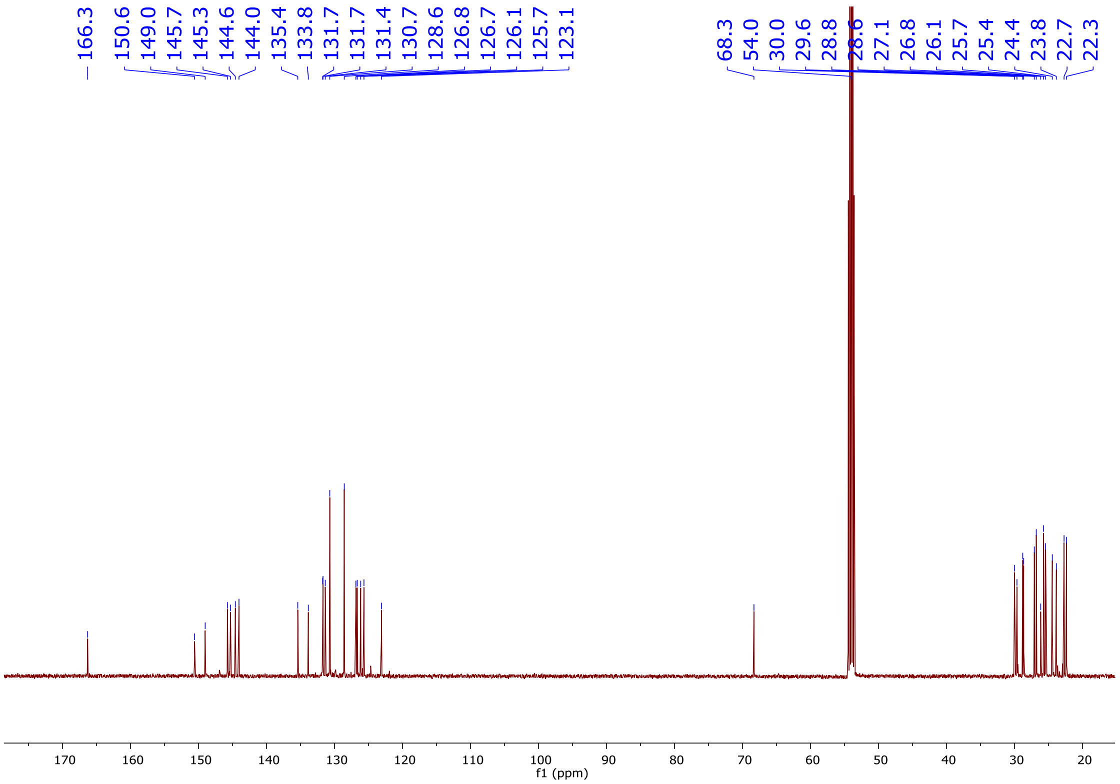

Figure S14. $^{13}C\{^{1}H\}$ NMR (125 MHz, CD_2Cl_2, 298 K) spectrum of compound **5-Ph**.

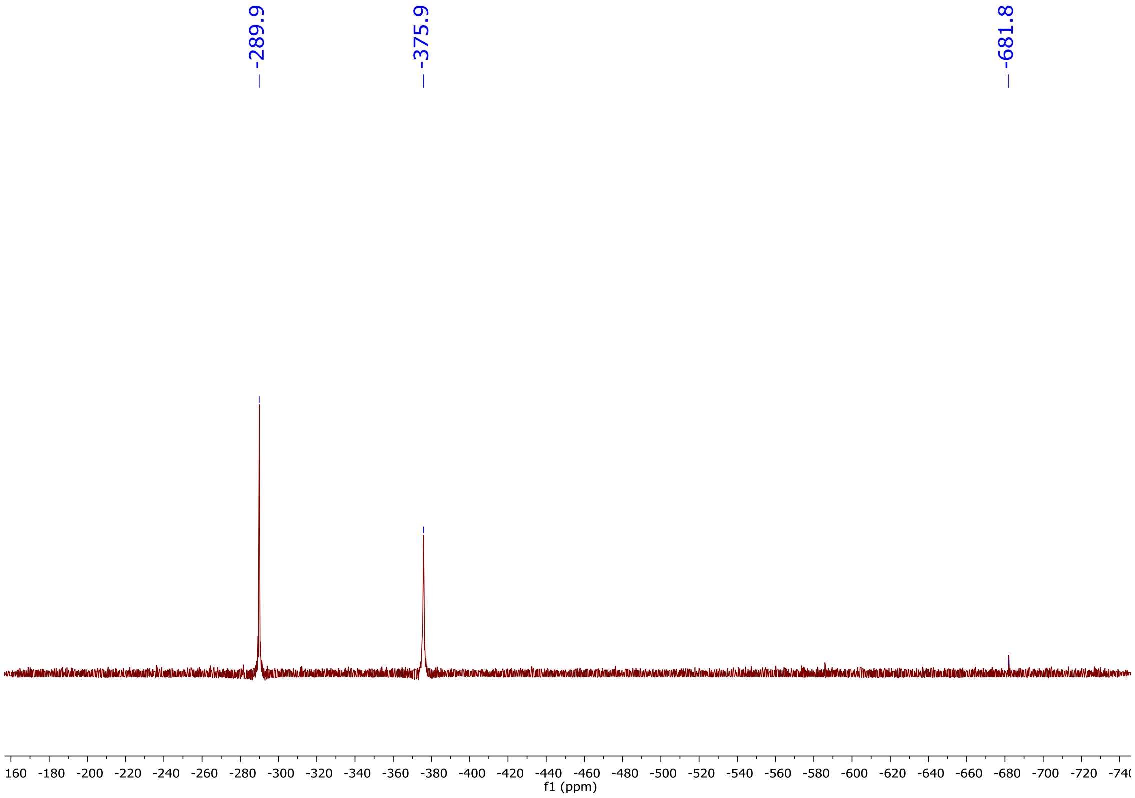

Figure S15. $^{119}Sn\{^{1}H\}$ NMR (186 MHz, CD_2Cl_2, 298 K) spectrum of compound **5-Ph**.

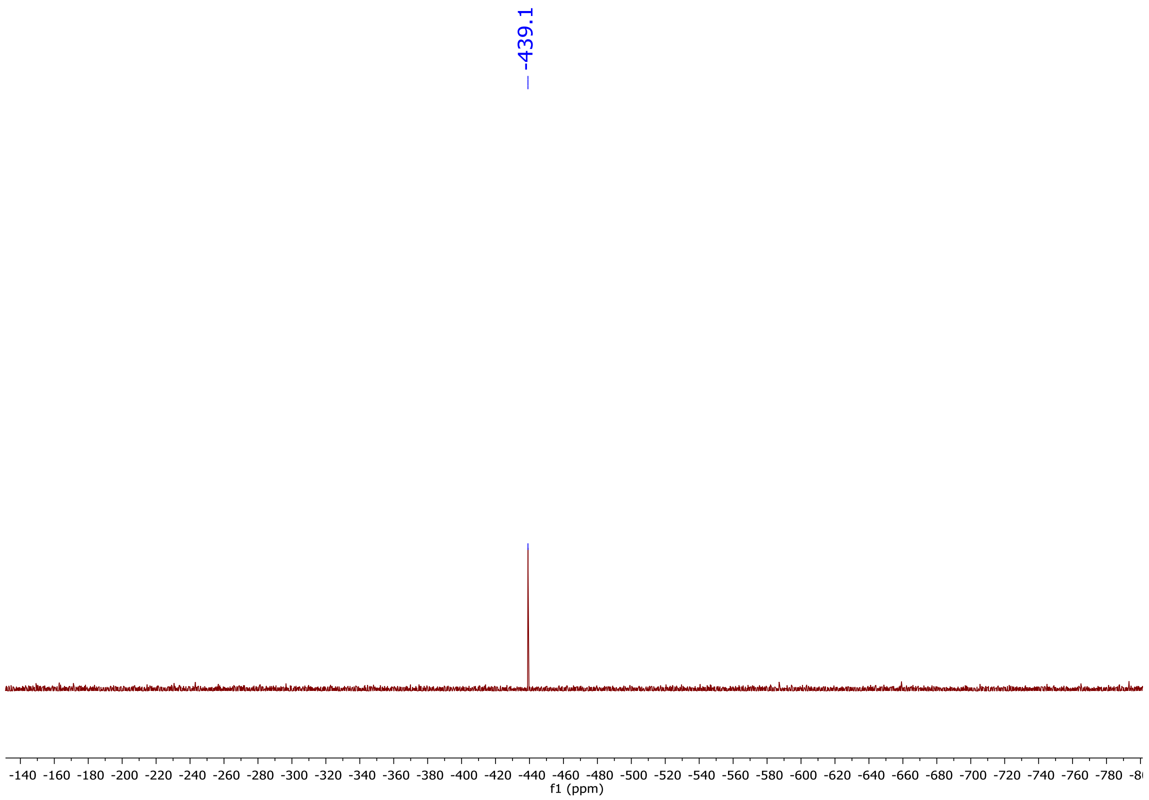

Figure S16. $^{77}Se\{^1H\}$ NMR (95 MHz, CD_2Cl_2, 298 K) spectrum of compound **5-Ph**.

Crystallographic Details

The single crystal data were examined on a Rigaku Supernova diffractometer using either MoKα (λ = 0.71073 Å) or CuKα (λ = 1.54184 Å) radiation. The crystals were kept at 100.0(1) K during data collection. Using Olex2,[3] the structure was solved with the ShelXT[4] structure solution program using Intrinsic Phasing and refined with the ShelXL[5] refinement package using Least Squares minimization. Hydrogen atoms were taken into account using a riding model. Crystals of **3-Ph** contain four solvent dcm molecules, two are disordered, one over three positions (0.61 0.18 0.21), one over two sites (88:12). The anion of **3-DMP** was modelled as a mixed crystal of Cl^- and $SnCl3^-$, the ratio refined to 21:79. The heavy residual electron density peaks near of these positions of the anions could not be modelled reasonably; it seems to be a complex problem of absorption effects and probably more anion position. No better model could be found. As solvent two non-disordered dcm molecules were modelled and one disordered one. Additionally, heavy disordered solvent molecules could not be refined reasonably, therefore, a solvent mask was calculated and 137 electrons were found in a volume of 542 $Å^3$ in three voids. This is consistent with the presence of 1.5 dcm molecules per formula unit, which account for 63.0 electrons. The sum formula and subsequent items count in these solvent molecules. The asymmetric unit of **4-Ph** contains besides two non-disordered thf molecules two heavy disordered ones. Therefore, a solvent mask was calculated and 168 electrons were found in a volume of 741 $Å^3$ in one void. This is consistent with the presence of

four thf molecules unit cell which account for 160.0 electrons. The asymmetric unit of **5-Ph** additionally contains three heavily disordered thf solvent molecules, which could not be refined reasonably. Therefore, a solvent mask was calculated and 257 electrons were found in a volume of 1063 Å^3 in two voids. This is consistent with the presence of three thf molecules per asymmetric unit which account for 120.0 electrons.

Details of the X-ray investigation are given in Table S1, and S2. CCDC 1986227-1986230 contains the supplementary crystallographic data for this paper. These data can be obtained free of charge from The Cambridge Crystallographic Data Centre via www.ccdc.cam.ac.uk/conts/retrieving.html.

Table S1. Crystal data and structure refinement for compounds **3-Ph** and **3-DMP**.

	**3-Ph**	**3-DMP**
Empirical formula	$C_{103}H_{125}Cl_{11}N_6Sn_3$	$C_{109.5}Cl_{11.57}H_{141}N_9Sn_{2.79}$
Formula weight	2193.10	2324.61
Temperature/K	100.00(1)	100.0(1)
Crystal system	monoclinic	triclinic
Space group	$P2_1/n$	P-1
a/Å	14.22611(10)	15.5105(2)
b/Å	36.93270(19)	18.22736(19)
c/Å	20.43246(12)	22.2468(3)
α/°	90	73.8678(10)
β/°	104.3262(6)	72.0895(11)
γ/°	90	89.0945(9)
Volume/$Å^3$	10401.55(11)	5732.34(13)
Z	4	2
$\rho_{calc}g/cm^3$	1.400	1.347
μ/mm^{-1}	1.045	0.922
F(000)	4488.0	2394.0
Crystal size/mm^3	0.33 × 0.175 × 0.161	0.301 × 0.23 × 0.174
Radiation/Å	Mo Kα (λ = 0.71073)	Mo Kα (λ = 0.71073)
2Θ range for data collection/°	3.156 to 63.882	3.452 to 64.388
Index ranges	-20 ≤ h ≤ 20, -54 ≤ k ≤ 54, -30 ≤ l ≤ 30	-23 ≤ h ≤ 22, -26 ≤ k ≤ 26, -32 ≤ l ≤ 33
Reflections collected	623888	159172
Independent reflections	34756 [R_{int} = 0.0851, R_{sigma} = 0.0381]	37502 [R_{int} = 0.0365, R_{sigma} = 0.0358]
Reflections with $I > 2\sigma(I)$	29202	30752
Data/restraints/parameters	34756/22/1163	37502/22/1229
Goodness-of-fit on F^2	1.083	1.090
Final R indexes [$I > 2\sigma(I)$]	R_1 = 0.0464, wR_2 = 0.1000	R_1 = 0.0505, wR_2 = 0.1238
Final R indexes [all data]	R_1 = 0.0610, wR_2 = 0.1056	R_1 = 0.0650, wR_2 = 0.1312
CCDC	1986227	1986228

Table S2. Crystal data and structure refinement for compounds **4-Ph** and **5-Ph**.

	**4-Ph**	**5-Ph**
Empirical formula	$C_{230}Cl_6H_{298}N_{12}O_8S_2Sn_5$	$C_{224}Cl_{10}H_{286}N_{12}O_6Se_2Sn_5$
Formula weight	4229.06	4348.50
Temperature/K	100.01(10)	100.0(1)
Crystal system	triclinic	triclinic
Space group	P-1	P-1
a/Å	14.6349(6)	14.6028(7)
b/Å	16.6803(7)	16.6496(7)
c/Å	22.6379(7)	22.6762(10)
α/°	95.408(3)	95.616(4)
β/°	92.127(3)	91.775(4)
γ/°	105.590(4)	105.462(4)
Volume/Å^3	5288.0(4)	5278.8(4)
Z	1	1
ρ_{calc}g/cm^3	1.328	1.368
μ/mm^{-1}	5.971	1.113
F(000)	2210	2250.0
Crystal size/mm^3	0.125 × 0.087 × 0.016	0.156 × 0.098 × 0.082
Radiation/Å	Cu Kα (λ = 1.54184)	Mo Kα (λ = 0.71073)
2Θ range for data collection/°	5.534 to 148.4	3.288 to 52.044
Index ranges	-17 ≤ h ≤ 17, -20 ≤ k ≤ 20, -28 ≤ l ≤ 23	-18 ≤ h ≤ 18, -20 ≤ k ≤ 20, -27 ≤ l ≤ 27
Reflections collected	39363	79471
Independent reflections	20857 [R_{int} = 0.1045, R_{sigma} = 0.1311]	20790 [R_{int} = 0.0840, R_{sigma} = 0.0915]
Reflections with $I > 2\sigma(I)$	14339	14856
Data/restraints/parameters	20857/81/1108	20790/64/1121
Goodness-of-fit on F^2	0.975	1.010
Final R indexes [$I > 2\sigma(I)$]	R_1 = 0.0614, wR_2 = 0.1375	R_1 = 0.0492, wR_2 = 0.0981
Final R indexes [all data]	R_1 = 0.0954, wR_2 = 0.1564	R_1 = 0.0759, wR_2 = 0.1099
CCDC	1986229	1986230

Selected bond lengths [Å] and angles [°] of 3-Ph, 3-DMP, 4-Ph and 5-Ph

3-Ph: Sn1−C2 2.263(2), Sn1−C35 2.248(2), Sn1−C68 2.246(2), Sn2−C3 2.266(2), Sn2−C36 2.259(2), Sn2−C69 2.269(2); C35−Sn1−C2 88.0(1), C68−Sn1−C2 86.9(1), C68−Sn1−C35 90.0(1), C3−Sn2−C69 87.6(1), C36−Sn2−C3 88.6(1), C36−Sn2−C69 89.1(1).

3-DMP: Sn1−C1 2.253(2), Sn1−C36 2.241(3), Sn1−C71 2.252(3), Sn2−C2 2.259(3), Sn2−C37 2.242(2), Sn2−C72 2.243(3); C36−Sn1−C1 87.4(1), C36−Sn1−C71 90.1(1), C71−Sn1−C1 88.5(1), C37−Sn2−C2 88.4(1), C37−Sn2−C72 89.9 (1), C72−Sn2−C2 87.7(1).

4-Ph: Sn1−S1 2.262(1), Sn1−C2 2.192(5), Sn1−C35 2.200(6), Sn1−C68 2.187(5), Sn2−C3 2.261(5), Sn2−C36 2.271(5), Sn2−C69 2.262(5); C2−Sn1−S1 124.0(2), C35−Sn1−S1 119.8(2), C35−Sn1−S1 119.8(2), C2−Sn1−C35 94.3(2), C68−Sn1−C2 95.4(2), C68−Sn1−C35 95.3(2), C3−Sn2−C36 88.7(2), C3−Sn2−C69 88.8(2), C69−Sn2−C36 88.1(2).

5-Ph: Sn1−Se1 2.388(1), Sn1−C1 2.195(4), Sn1−C34 2.200(4), Sn1−C67 2.187(4), Sn2−C2 2.259(4), Sn2−C35 2.269(4), Sn2−C68 2.278(4) ; C1−Sn1−Se1 124.5(1), C34−Sn1−Se1 120.7(1), C67−Sn1−Se1 119.7(1), C1−Sn1−C34 94.8(1), C67−Sn1−C1 94.6(2), C67−Sn1−C34 95.6(2), C2−Sn2−C35 88.4(1), C2−Sn2−C68 88.4(1), C35−Sn2−C68 87.8(1), C51−C52−C53 120.5(4), C1−N1−C4 123.6(3).

Computational Details

All computations were performed using Gaussian 16 program suite.[6] The geometry optimizations were carried out using the B3LYP/6-31G(d) level of theory. LanL2DZ was used as pseudopotential for the core orbitals and as basis sets for the valence orbitals of Sn. [7-9] The integral accuracy was internally set to 10^{-6} E_H with an ultrafine grid. The coordinates used for the geometry optimizations of compounds **3-Ph**, **4-Ph**, and **5-Ph** were obtained from single-crystal X-ray diffraction studies. Frequency calculations were carried out for all the optimized geometries to characterize the stationary points as minima. It was always used C_1 symmetry. The aforementioned level of theory and optimized coordinates were used for performing the Weinhold's natural bond orbital (NBO) analysis.[10]

The Wiberg Bond Indices (WBI)[11] and NPA[12] atomic partial charges were calculated at the same level of theory using the NBO 3.1 interface of Gaussian.[13]

Table S3. Wiberg bond indices (WBIs) of compounds **3-Ph**, **4-Ph**, and **5-Ph** calculated at B3LYP/6-31G(d) level of theory.

**3-Ph**		**4-Ph**		**5-Ph**	
**bond**	**WBI**	**bond**	**WBI**	**bond**	**WBI**
Sn1- C10	0.55	Sn1-C11	0.59	Sn12-C9	0.52
Sn1-C82	0.55	Sn1-C83	0.59	Sn12-C13	0.52
Sn1-C154	0.55	Sn1-C155	0.57	Sn12-C70	0.52
Sn2-C11	0.55	Sn2-C12	0.55	Sn18-C10	0.60
Sn2-C83	0.55	Sn2-C84	0.55	Sn18-C14	0.62
Sn2-C155	0.55	Sn2-C156	0.55	Sn18-C66	0.62
		S3-Sn1	1.09	Se19-Sn18	0.96

Table S4. Natural population analysis (NPA) atomic charges of compounds **3-Ph**, **4-Ph**, and **5-Ph** calculated at B3LYP/6-31G(d) level of theory.

**3-Ph**		**4-Ph**		**5-Ph**	
**Atom**	**charge**	**Atom**	**charge**	**Atom**	**charge**
Sn1	0.94	Sn1	1.79	Sn1	1.74
Sn2	0.94	Sn2	1.55	Sn2	1.30
C10	-0.29	S3	-0.56	Se19	-0.27
C82	-0.29	C11	-0.32	C9	-0.29
C154	-0.29	C83	-0.33	C10	-0.37
C11	-0.29	C155	-0.31	C13	-0.29
C83	-0.29	C12	-0.329	C14	-0.34
C155	-0.29	C84	0.11	C66	-0.34
		C156	-0.31	C70	-0.29

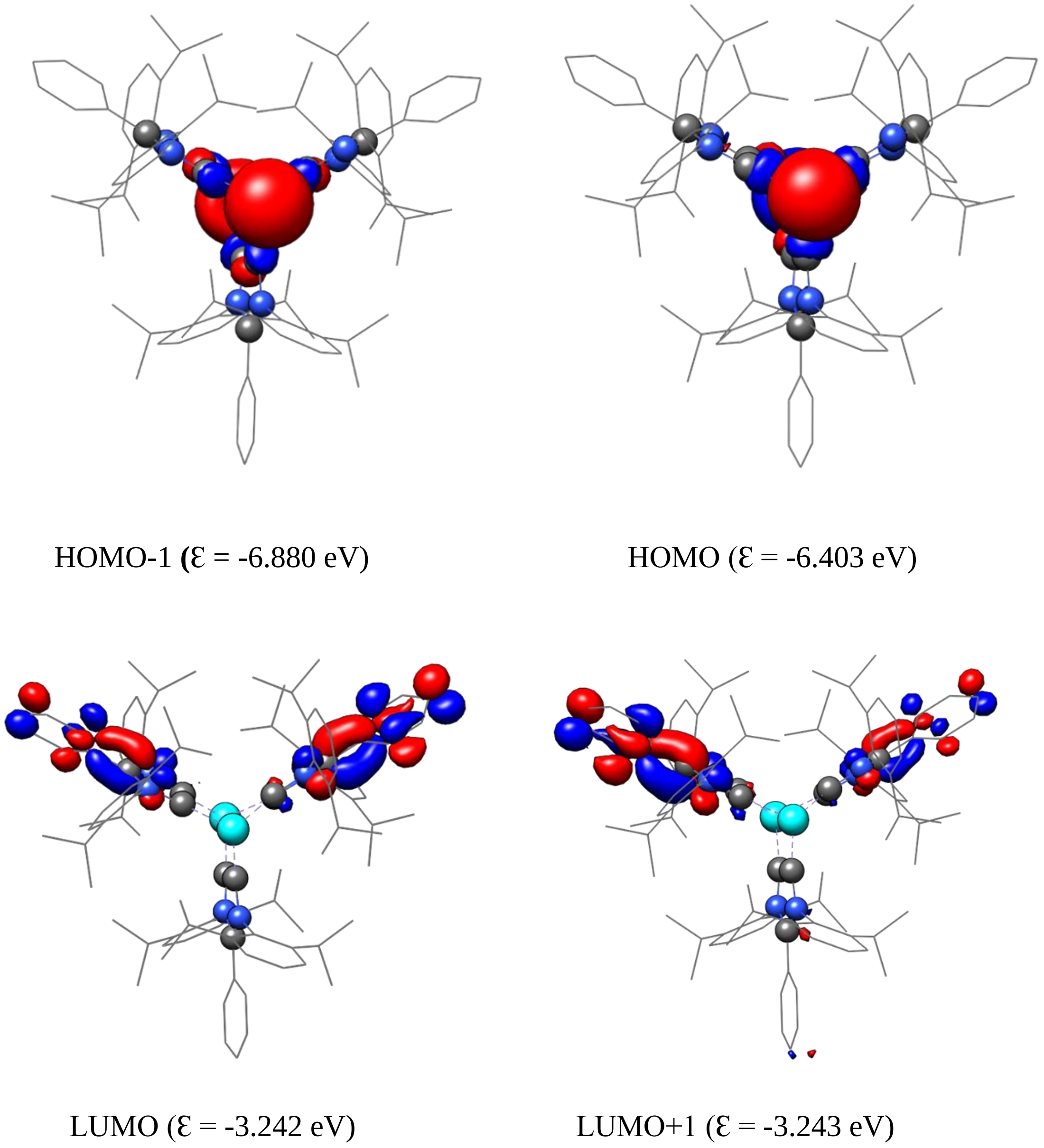

Figure S17. Selected molecular orbitals (from HOMO−1 to LUMO+1) of compound **3-Ph**, calculated at B3LYP/6-31G(d). The isovalue was arbitrarily chosen to be 0.04. Hydrogen atoms were omitted for clarity.

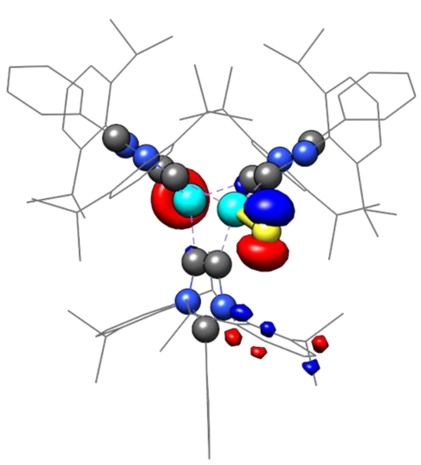

HOMO (Ɛ = -12.047 eV)

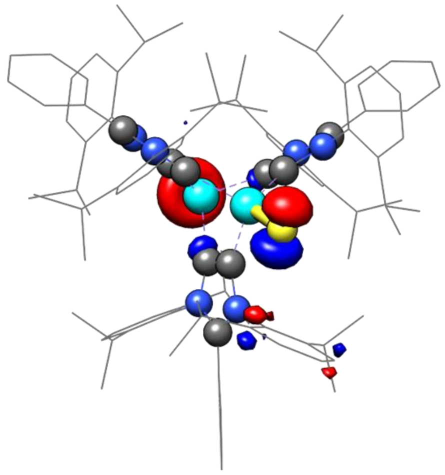

LUMO (Ɛ = -11.536 eV)

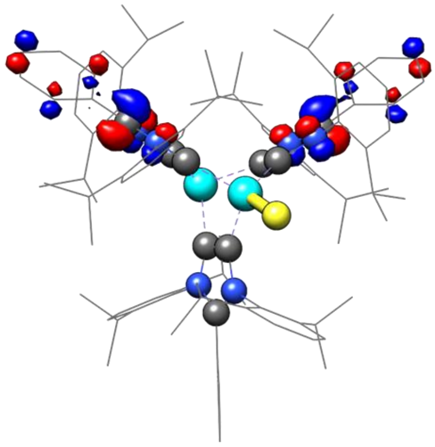

LUMO+1 (Ɛ = -7.932 eV)

Figure S18. Selected molecular orbitals (from HOMO to LUMO+1) of compound **4-Ph** calculated at B3LYP/6-31G(d). The isovalue was arbitrarily chosen to be 0.04. Hydrogen atoms were omitted for clarity.

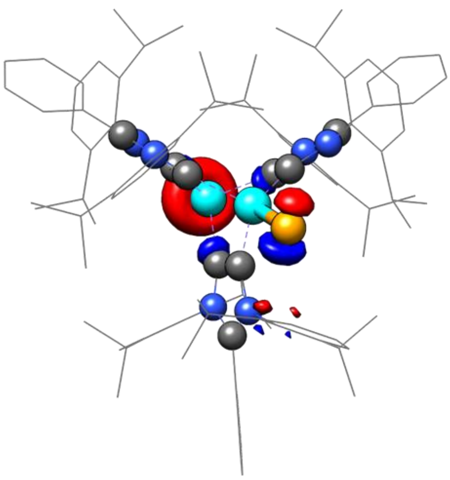

HOMO (Ɛ = -11.789 eV)

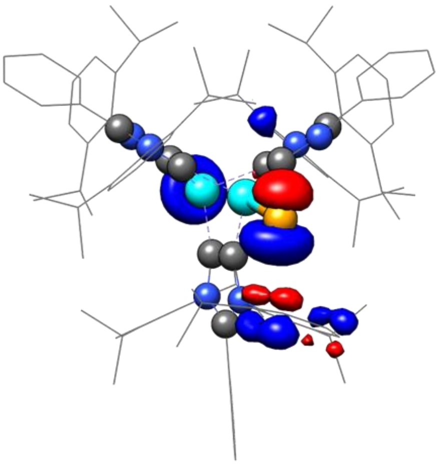

LUMO (Ɛ = -11.337 eV)

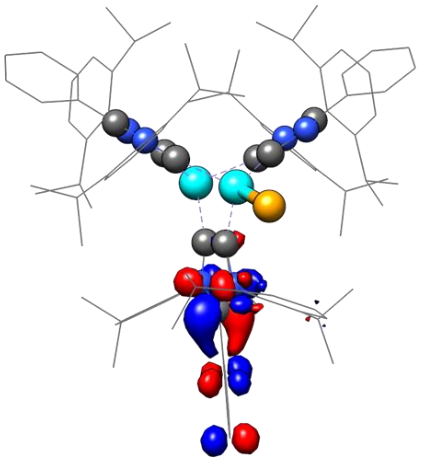

LUMO+1 (Ɛ = -7.942 eV)

Figure S19. Selected molecular orbitals (from HOMO to LUMO+1) of compound **5-Ph** calculated at B3LYP/6-31G(d). The isovalue was arbitrarily chosen to be 0.04. Hydrogen atoms were omitted for clarity.

Cartesian Coordinates for the Optimized Geometries of Compounds 3-Ph, 4-Ph, and 5-Ph:

<u>3-Ph</u>

Sn	9.89564	15.07331	7.27801
Sn	8.24789	13.00028	4.23176
N	11.7334	15.65574	4.62398
N	11.11088	14.28077	3.05918
N	9.87881	11.90035	8.20097
N	8.72516	10.8451	6.69303
N	6.93051	16.4458	6.74019
N	6.21942	15.53379	4.89564
C	12.00619	15.25864	3.35156
C	10.64283	14.92861	5.14654
C	10.24585	14.06608	4.16128
C	12.31134	16.80479	5.28813
C	13.09911	16.59915	6.43135
C	13.63698	17.73497	7.04484
H	14.15849	17.63555	7.80844
C	13.41035	19.00276	6.54093
H	13.79591	19.74064	6.9554
C	12.61698	19.17915	5.42877
H	12.46724	20.03777	5.10428
C	12.03121	18.08203	4.77806
C	13.43698	15.21447	6.97549
H	12.72579	14.59979	6.69834
C	14.75419	14.71425	6.37232
H	14.64004	14.56918	5.43023
H	15.00696	13.89002	6.79469
H	15.44062	15.37021	6.51422
C	13.52121	15.17919	8.51004

H	14.31868	15.62996	8.79718
H	13.54421	14.26699	8.80837
H	12.75339	15.61854	8.88286
C	11.08397	18.33421	3.61552
H	10.84626	17.46651	3.2269
C	11.73789	19.18233	2.51334
H	11.95913	20.04796	2.86452
H	11.12602	19.28124	1.78025
H	12.53703	18.7476	2.20676
C	9.797	18.99017	4.12589
H	9.34421	18.38702	4.71986
H	9.2259	19.19574	3.38211
H	10.01441	19.79874	4.59547
C	11.234	13.38316	1.91923
C	12.34655	12.51889	1.88082
C	12.46358	11.66254	0.78375
H	13.19441	11.09012	0.7278
C	11.51183	11.64961	-0.22256
H	11.60636	11.06895	-0.94283
C	10.42525	12.49478	-0.15933
H	9.78892	12.47011	-0.8371
C	10.25878	13.39475	0.91025
C	13.41491	12.4618	2.95055
H	13.23654	13.1784	3.59481
C	14.82035	12.69263	2.35866
H	15.05792	11.94961	1.79912
H	15.45841	12.7734	3.07138
H	14.81949	13.49861	1.83711
C	13.3636	11.13004	3.69971
H	12.52534	11.05477	4.16152

H	14.08305	11.09189	4.33419
H	13.4502	10.40727	3.07387
C	9.07748	14.34171	0.8684
H	9.01302	14.79805	1.73327
C	9.26192	15.39979	-0.2297
H	9.9855	15.9844	0.0075
H	8.45431	15.91079	-0.32061
H	9.46123	14.9655	-1.06233
C	7.76312	13.58736	0.61468
H	7.73212	13.29714	-0.29989
H	7.02067	14.16986	0.79089
H	7.71538	12.8237	1.1945
C	13.06286	15.78648	2.4794
C	14.27098	16.24459	3.01624
H	14.40567	16.21523	3.93595
C	15.27229	16.74315	2.19096
H	16.07268	17.03924	2.56055
C	15.08272	16.80043	0.8232
H	15.7534	17.13419	0.27211
C	13.88817	16.35805	0.27672
H	13.75953	16.40001	-0.64338
C	12.87526	15.84967	1.09403
H	12.07778	15.55384	0.718
C	9.37667	10.68869	7.86959
C	9.53488	12.85524	7.21805
C	8.81419	12.18994	6.26328
C	10.81046	12.14392	9.29492
C	10.3999	12.96231	10.36723
C	11.31874	13.17992	11.3941
H	11.07634	13.71378	12.116

C	12.5863	12.61713	11.36157
H	13.18759	12.78429	12.05106
C	12.95445	11.80842	10.30435
H	13.80286	11.42757	10.29778
C	12.0813	11.54904	9.2438
C	8.99095	13.53927	10.48357
H	8.60995	13.58533	9.58184
C	8.97621	14.95335	11.07198
H	9.18888	14.91298	12.00725
H	8.10407	15.33812	10.95843
H	9.62649	15.49519	10.61904
C	8.09429	12.61824	11.32732
H	7.93795	11.79967	10.85077
H	7.25633	13.05497	11.49673
H	8.52878	12.42501	12.1613
C	12.57113	10.64681	8.11373
H	11.82905	10.51293	7.48778
C	13.72155	11.30536	7.35225
H	13.40059	12.09199	6.90523
H	14.07068	10.68906	6.70428
H	14.41579	11.5486	7.96905
C	13.0039	9.26882	8.64322
H	13.79249	9.36628	9.18196
H	13.19159	8.68631	7.9036
H	12.29766	8.89399	9.17457
C	7.91771	9.83075	6.03846
C	8.34655	9.33965	4.78784
C	7.52004	8.40208	4.15382
H	7.76007	8.0763	3.31646
C	6.35494	7.95109	4.7472

H	5.82468	7.32457	4.30994
C	5.97631	8.42782	5.98653
H	5.19629	8.10754	6.37881
C	6.7421	9.38309	6.66454
C	9.67786	9.71001	4.14406
H	9.9529	10.58174	4.49742
C	9.57676	9.82772	2.61874
H	9.46111	8.95437	2.23737
H	10.38077	10.22338	2.27433
H	8.82511	10.37912	2.38945
C	10.75879	8.68029	4.52329
H	10.99604	8.7908	5.44692
H	11.53532	8.81488	3.97513
H	10.41869	7.79339	4.38418
C	6.24522	9.87939	8.02298
H	6.88569	10.53845	8.36331
C	4.88092	10.56338	7.878
H	4.96609	11.32911	7.30529
H	4.56974	10.84304	8.74204
H	4.25259	9.94627	7.49588
C	6.14583	8.73451	9.03862
H	5.49202	8.09798	8.74034
H	5.88374	9.08618	9.89259
H	6.99952	8.30277	9.11868
C	9.51652	9.44473	8.64055
C	9.62633	8.20552	7.99985
H	9.60125	8.16028	7.07129
C	9.77293	7.04037	8.74715
H	9.85311	6.21936	8.31771
C	9.79938	7.10415	10.13313

H	9.88817	6.32391	10.63136
C	9.69379	8.32777	10.7779
H	9.71561	8.36651	11.70683
C	9.55531	9.49717	10.03711
H	9.48832	10.31634	10.47223
C	5.95103	16.49263	5.80725
C	7.85438	15.42661	6.41029
C	7.40686	14.84913	5.24353
C	6.76214	17.01412	8.07235
C	7.52601	18.10559	8.50076
C	7.2875	18.58498	9.79254
H	7.77507	19.31462	10.10038
C	6.34854	18.00628	10.6251
H	6.20729	18.34776	11.47853
C	5.61741	16.9133	10.18189
H	4.99206	16.5224	10.7485
C	5.80317	16.3897	8.90129
C	8.58365	18.81245	7.67271
H	8.62508	18.38203	6.79324
C	8.25181	20.29858	7.46824
H	7.38319	20.38035	7.06771
H	8.90854	20.69683	6.89231
H	8.25634	20.74717	8.31698
C	9.96504	18.71617	8.32071
H	9.96219	19.1947	9.15291
H	10.62122	19.0989	7.73374
H	10.17915	17.7944	8.48233
C	5.01471	15.15009	8.48688
H	5.11844	15.03468	7.51926
C	3.52278	15.26628	8.791

H	3.39477	15.34672	9.73902
H	3.06923	14.48257	8.47219
H	3.16643	16.04268	8.35299
C	5.59666	13.91861	9.1736
H	6.49951	13.78332	8.87669
H	5.06802	13.14863	8.95162
H	5.58871	14.04883	10.12469
C	5.4499	15.29018	3.68837
C	4.73629	14.08137	3.59348
C	4.01248	13.86989	2.41485
H	3.51817	13.08773	2.32107
C	4.01305	14.79369	1.38579
H	3.53475	14.62288	0.60672
C	4.72339	15.96822	1.51592
H	4.71456	16.58437	0.81938
C	5.45727	16.25382	2.67256
C	4.66319	13.03913	4.7093
H	5.42392	13.18006	5.31082
C	3.37044	13.22582	5.51754
H	3.38402	14.08307	5.94939
H	3.30486	12.53455	6.18046
H	2.61447	13.17648	4.9279
C	4.73517	11.60304	4.17241
H	3.93152	11.40212	3.68726
H	4.82727	10.99121	4.90644
H	5.49175	11.51754	3.5877
C	6.20878	17.57705	2.74282
H	6.59686	17.66303	3.63858
C	5.24991	18.75661	2.51759
H	4.90979	18.72569	1.62039

H	5.7208	19.58216	2.65304
H	4.52016	18.69888	3.13865
C	7.34857	17.61454	1.71955
H	8.02183	16.97649	1.96696
H	7.73279	18.49409	1.70061
H	7.00521	17.39652	0.84997
C	4.8501	17.46777	5.76387
C	3.54335	17.09349	5.42637
H	3.34596	16.20216	5.24913
C	2.53911	18.05684	5.35625
H	1.67104	17.80419	5.13835
C	2.82698	19.39661	5.61049
H	2.15271	20.03551	5.5648
C	4.12042	19.77336	5.93134
H	4.31694	20.66802	6.09217
C	5.12781	18.81444	6.01363
H	5.99287	19.07286	6.23679
H	17.17075	7.93952	7.29864
C	16.57459	6.89096	8.93321
H	16.90579	6.05225	8.63428
C	15.93136	6.97884	10.17304
C	15.05533	10.74814	10.15519
H	14.76051	10.72498	11.11047
C	13.83946	11.10319	9.28068
H	14.06115	10.9556	8.33755
H	13.60255	12.04428	9.41715
H	13.08051	10.53574	9.53051
C	16.13083	11.84152	10.00521
H	16.92988	11.5854	10.51151
H	15.78377	12.69061	10.35017

H	16.36281	11.94389	9.05859
C	15.83279	5.70698	11.00505
H	15.28107	5.90619	11.81493
C	15.1505	4.56958	10.22832
H	14.26533	4.86413	9.92809
H	15.05426	3.78775	10.81131
H	15.69686	4.33169	9.45033
C	17.21424	5.25424	11.46991
H	17.78796	5.10279	10.68996
H	17.13057	4.42192	11.98042
H	17.61119	5.9473	12.03779

3a

Sn	0.00666	0.00353	-1.72353
Sn	0.01506	-0.0277	1.81522
S	0.46822	-0.18513	-3.97988
N	-0.72706	3.1859	-1.019
N	-1.03028	3.0399	1.15652
N	-2.45244	-2.16234	-1.04733
N	-2.15027	-2.42334	1.11851
N	3.09344	-0.93885	-1.04326
N	3.22752	-0.69542	1.14198
C	-1.09008	3.89154	0.09105
C	-0.44327	1.85668	-0.65307
C	-0.61514	1.77709	0.71193
C	-0.43604	3.77605	-2.3281
C	0.69304	4.62662	-2.43903
C	0.96857	5.16314	-3.70483
H	1.82284	5.82181	-3.82396
C	0.16706	4.88542	-4.80494

H	0.40035	5.31789	-5.77318
C	-0.95	4.06872	-4.65722
H	-1.58565	3.88359	-5.51644
C	-1.28648	3.49611	-3.42316
C	1.60052	5.0421	-1.28004
H	1.22409	4.60075	-0.35201
C	1.60976	6.57579	-1.09625
H	2.10072	7.07377	-1.93878
H	2.16881	6.83944	-0.19186
H	0.6008	6.9851	-1.00766
C	3.04195	4.53188	-1.45883
H	3.0801	3.43853	-1.48051
H	3.66449	4.87849	-0.62767
H	3.49041	4.90978	-2.38371
C	-2.5755	2.67591	-3.34031
H	-2.59357	2.15386	-2.37568
C	-3.82898	3.58078	-3.40354
H	-3.91565	4.24432	-2.53842
H	-4.73422	2.96674	-3.43628
H	-3.81694	4.2012	-4.30579
C	-2.67719	1.61679	-4.45611
H	-2.82055	2.07846	-5.43864
H	-3.53654	0.96365	-4.27709
H	-1.77683	0.99403	-4.52018
C	-1.51387	3.34261	2.50446
C	-0.57883	3.48734	3.55741
C	-1.08238	3.78559	4.83129
H	-0.39128	3.91143	5.65839
C	-2.4438	3.95475	5.05412
H	-2.80734	4.20258	6.04682

C	-3.3375	3.81992	3.99657
H	-4.39735	3.96655	4.17808
C	-2.90555	3.51019	2.69944
C	0.93803	3.39127	3.39487
H	1.16663	3.11488	2.35804
C	1.63066	4.74444	3.67413
H	1.30469	5.52582	2.98103
H	2.71536	4.64139	3.57037
H	1.42711	5.09356	4.69138
C	1.53409	2.31116	4.31862
H	1.3937	2.56001	5.37545
H	2.6075	2.19851	4.14382
H	1.05908	1.33155	4.16037
C	-3.9669	3.39922	1.60469
H	-3.47649	3.18676	0.64882
C	-4.75721	4.716	1.44469
H	-5.36863	4.92293	2.32928
H	-5.43733	4.64097	0.58939
H	-4.09892	5.57322	1.28444
C	-4.93739	2.2352	1.88348
H	-4.42083	1.26939	1.88293
H	-5.71696	2.20527	1.11628
H	-5.43792	2.35318	2.85032
C	-1.46395	5.31432	0.13997
C	-1.13373	6.10844	1.25644
H	-0.59135	5.68456	2.08894
C	-1.49049	7.45339	1.30118
H	-1.22001	8.04913	2.16736
C	-2.18618	8.03477	0.23808
H	-2.46594	9.0831	0.27667

C	-2.51576	7.25982	-0.87643
H	-3.05696	7.70058	-1.70777
C	-2.15563	5.91568	-0.92963
H	-2.41851	5.33839	-1.80299
C	-2.90065	-2.8362	0.05238
C	-1.40591	-1.29807	-0.67172
C	-1.23139	-1.45599	0.68725
C	-3.1156	-2.1763	-2.35536
C	-4.37954	-1.54874	-2.46208
C	-5.01395	-1.5902	-3.71302
H	-5.98716	-1.12392	-3.82745
C	-4.43155	-2.22388	-4.8016
H	-4.94393	-2.24661	-5.75868
C	-3.19143	-2.84167	-4.66178
H	-2.75715	-3.34142	-5.51898
C	-2.50034	-2.84577	-3.44283
C	-5.12334	-0.86192	-1.31598
H	-4.53117	-0.94809	-0.39859
C	-5.31565	0.64125	-1.58936
H	-4.35718	1.16461	-1.656
H	-5.89857	1.09681	-0.78329
H	-5.86161	0.81182	-2.52296
C	-6.49465	-1.52194	-1.0525
H	-7.18164	-1.35676	-1.88898
H	-6.95496	-1.08079	-0.1617
H	-6.4107	-2.59981	-0.89673
C	-1.1783	-3.61623	-3.35064
H	-0.53675	-3.1021	-2.62443
C	-0.41468	-3.66247	-4.68823
H	-0.88086	-4.35895	-5.39332

H	0.6035	-4.02299	-4.51273
H	-0.3468	-2.67954	-5.15875
C	-1.38366	-5.0658	-2.85372
H	-1.7187	-5.11793	-1.81429
H	-0.44357	-5.62192	-2.92382
H	-2.11809	-5.58881	-3.47658
C	-2.12257	-3.06862	2.43394
C	-2.65623	-2.39143	3.55632
C	-2.60976	-3.05217	4.79209
H	-3.01865	-2.56255	5.67006
C	-2.08236	-4.33228	4.91396
H	-2.07554	-4.83005	5.87895
C	-1.57611	-4.97814	3.79085
H	-1.17669	-5.98209	3.89269
C	-1.57586	-4.37019	2.52747
C	-3.31846	-1.01522	3.50896
H	-3.2752	-0.63879	2.47937
C	-2.58601	-0.00981	4.41765
H	-2.62471	-0.31077	5.46976
H	-3.02936	0.98619	4.33625
H	-1.51861	0.0714	4.15619
C	-4.80819	-1.07371	3.91777
H	-5.39533	-1.71517	3.25427
H	-5.2465	-0.07167	3.8814
H	-4.92758	-1.45013	4.93851
C	-1.00526	-5.16587	1.35458
H	-1.09079	-4.56765	0.44159
C	0.49323	-5.46506	1.55518
H	1.08302	-4.54429	1.62474
H	0.87332	-6.05166	0.71289

H	0.66849	-6.04995	2.4643
C	-1.78324	-6.48032	1.12988
H	-1.65109	-7.16962	1.97042
H	-1.41193	-6.98611	0.23199
H	-2.85499	-6.30532	1.00535
C	-4.01671	-3.79742	0.10712
C	-4.80922	-3.89183	1.26964
H	-4.60712	-3.25953	2.12039
C	-5.87118	-4.78839	1.34119
H	-6.46904	-4.83661	2.24597
C	-6.169	-5.61603	0.25631
H	-6.99496	-6.3183	0.31405
C	-5.3998	-5.52784	-0.90583
H	-5.62296	-6.16265	-1.75761
C	-4.34054	-4.62725	-0.98615
H	-3.77322	-4.57992	-1.90197
C	3.93251	-1.06555	0.02962
C	1.82302	-0.5114	-0.59012
C	1.9187	-0.37439	0.75962
C	3.37674	-1.36241	-2.40314
C	3.48825	-2.74113	-2.67207
C	3.75252	-3.11715	-3.99939
H	3.86017	-4.1709	-4.23518
C	3.88863	-2.17739	-5.02216
H	4.08896	-2.50539	-6.03742
C	3.77917	-0.82733	-4.7368
H	3.89551	-0.10572	-5.53524
C	3.53092	-0.36324	-3.42476
C	3.37876	-3.85077	-1.62614
H	3.24426	-3.39819	-0.63918

C	2.15086	-4.74163	-1.88458
H	1.22066	-4.16972	-1.82335
H	2.11044	-5.54355	-1.14089
H	2.19682	-5.21302	-2.87206
C	4.65688	-4.7152	-1.57163
H	4.79066	-5.2919	-2.49298
H	4.58056	-5.43386	-0.74896
H	5.5556	-4.11348	-1.41747
C	3.61842	1.13977	-3.1532
H	2.92629	1.38207	-2.33776
C	3.24571	2.00861	-4.36945
H	4.02484	1.97993	-5.13858
H	3.15554	3.04976	-4.05027
H	2.29584	1.70933	-4.81694
C	5.03958	1.54866	-2.68825
H	5.29534	1.15667	-1.70154
H	5.10159	2.63975	-2.63477
H	5.79871	1.2128	-3.40331
C	3.80033	-0.43875	2.46466
C	3.46935	-1.29636	3.54012
C	4.02801	-1.00623	4.79226
H	3.7994	-1.6488	5.63639
C	4.88836	0.0705	4.97294
H	5.31951	0.26604	5.9501
C	5.20667	0.88804	3.89319
H	5.89018	1.7179	4.04133
C	4.67576	0.66316	2.61589
C	2.58007	-2.53283	3.4213
H	2.21387	-2.60681	2.38991
C	1.35783	-2.43489	4.35406

H	1.65406	-2.41443	5.40798
H	0.69086	-3.28937	4.2115
H	0.78068	-1.516	4.17208
C	3.35591	-3.83343	3.72883
H	4.19299	-3.98848	3.04133
H	2.69023	-4.69771	3.63987
H	3.75702	-3.82768	4.74719
C	5.10017	1.60664	1.49108
H	4.62382	1.28725	0.55847
C	4.63379	3.05104	1.75643
H	3.54219	3.11684	1.81709
H	4.96981	3.70688	0.94701
H	5.05108	3.4435	2.68983
C	6.62813	1.57379	1.27243
H	7.16202	1.98606	2.13492
H	6.89543	2.18368	0.40264
H	6.99642	0.55814	1.10558
C	5.31881	-1.56336	0.01265
C	5.82054	-2.28309	1.11718
H	5.19347	-2.47174	1.9757
C	7.12437	-2.76993	1.12014
H	7.48426	-3.32549	1.98045
C	7.96307	-2.54827	0.02542
H	8.98233	-2.92207	0.0321
C	7.47763	-1.84771	-1.08101
H	8.11804	-1.67202	-1.93971
C	6.17063	-1.36723	-1.09406
H	5.82639	-0.83652	-1.96825

4a

C	-5.39552	-3.28024	-0.93612
C	-4.90418	-2.56897	0.17801
C	-5.74426	-2.4421	1.30371
C	-7.01196	-3.0173	1.31864
C	-7.47414	-3.73988	0.21657
C	-6.65989	-3.86473	-0.91104
C	-3.56416	-1.95299	0.17627
N	-2.77235	-1.72217	1.26538
C	-1.59638	-1.0535	0.87454
C	-1.67237	-0.90789	-0.48931
N	-2.88865	-1.47074	-0.91041
Sn	0.02173	0.01422	2.03058
C	1.68578	-0.85983	0.77965
C	1.59751	-1.05838	-0.5744
N	2.7267	-1.79989	-0.972
C	3.50352	-2.03749	0.12495
N	2.88019	-1.46902	1.19887
Sn	-0.02937	-0.02833	-1.57139
Se	-0.57402	-0.18931	-4.00089
C	2.90106	-2.43412	-2.27967
C	2.68419	-3.83046	-2.38235
C	2.83656	-4.41012	-3.64913
C	3.19467	-3.65695	-4.76266
C	3.42438	-2.2933	-4.62683
C	3.29089	-1.64602	-3.39007
C	4.77956	-2.77267	0.1452
C	5.15034	-3.55258	1.25845
C	6.35843	-4.24495	1.27036
C	7.22533	-4.17204	0.17739
C	6.87023	-3.40233	-0.93281

C	5.66051	-2.71297	-0.95183
C	3.46194	-1.34969	2.5369
C	2.92546	-2.1202	3.59779
C	3.52996	-1.99173	4.8559
C	4.61889	-1.15259	5.05971
C	5.12929	-0.41463	3.99698
C	4.57315	-0.49033	2.71292
C	2.35689	-4.75921	-1.21076
C	0.97111	-5.41571	-1.35609
C	3.64302	-0.16027	-3.31676
C	2.90048	0.68286	-4.37118
C	1.77502	-3.11937	3.46023
C	0.66545	-2.85798	4.49952
C	5.22716	0.33884	1.60734
C	5.10409	1.84756	1.89302
C	-2.98712	-2.3036	2.59504
C	-3.34076	-1.46391	3.67667
C	-3.53966	-2.07221	4.92389
C	-3.40389	-3.44484	5.09659
C	-3.06148	-4.24533	4.01249
C	-2.84452	-3.70446	2.73788
C	-3.33859	-1.34629	-2.28785
C	-4.38266	-0.46106	-2.59662
C	-4.68173	-0.24324	-3.96946
C	-3.9333	-0.81224	-4.97981
C	-2.9046	-1.7023	-4.6525
C	-2.61306	-2.05386	-3.29839
C	-5.21104	0.3038	-1.57186
C	-6.71829	-0.009	-1.7063
C	-1.72828	-3.28112	-3.0303

C	-2.58139	-4.49396	-2.58128
C	-3.56891	0.04434	3.57361
C	-5.05744	0.41196	3.76556
C	-2.49201	-4.66948	1.60624
C	-3.6043	-5.71702	1.38464
C	0.08635	1.89963	-0.56504
N	0.22096	3.24428	-0.96943
C	0.0938	4.04986	0.12739
N	-0.12275	3.23456	1.20587
C	-0.11024	1.89311	0.7884
C	0.20921	5.52064	0.15996
C	-0.11889	6.32249	-0.95389
C	-0.00388	7.70903	-0.89472
C	0.45133	8.33455	0.26757
C	0.79375	7.55506	1.37423
C	0.67223	6.16953	1.32379
C	0.66059	3.69049	-2.28904
C	2.00517	4.10586	-2.4343
C	2.40641	4.55373	-3.7013
C	1.52253	4.59428	-4.7727
C	0.20299	4.18894	-4.59895
C	-0.27353	3.73467	-3.36119
C	-0.56376	3.68519	2.52855
C	0.31646	3.59656	3.63483
C	-0.15499	4.0618	4.87052
C	-1.42847	4.60011	5.01136
C	-2.26955	4.67884	3.90603
C	-1.86723	4.22652	2.64269
C	3.0395	4.14611	-1.30755
C	4.22845	3.21174	-1.59734

C	-1.76215	3.39344	-3.22184
C	-2.41432	2.98902	-4.55758
C	1.75704	3.08704	3.57099
C	2.02042	1.98083	4.61366
C	-2.85239	4.37872	1.48457
C	-4.12501	3.53956	1.70947
C	-4.97068	1.82282	-1.68436
C	-0.87908	-3.72648	-4.23449
C	-2.71805	0.8193	4.60047
C	-1.14643	-5.37437	1.86274
C	3.43516	-5.85257	-1.04395
C	5.16298	0.07549	-3.49839
C	2.26688	-4.57803	3.60025
C	6.71259	-0.03765	1.41722
C	3.55589	5.58084	-1.06078
C	-2.58018	4.56061	-2.62333
C	2.77942	4.22755	3.78152
C	-3.2277	5.8575	1.24867
H	-5.49892	0.42358	-4.22265
H	-4.14961	-0.59542	-6.0209
H	-2.37777	-2.21567	-5.44715
H	-4.90182	0.00347	-0.56903
H	-7.12743	0.3553	-2.65444
H	-7.26395	0.49635	-0.90303
H	-6.9221	-1.07994	-1.6345
H	-3.91908	2.0779	-1.52301
H	-5.56802	2.34052	-0.92751
H	-5.2737	2.21048	-2.66267
H	-1.05114	-3.04114	-2.20204
H	-3.01433	-4.36631	-1.58848

H	-1.94322	-5.38167	-2.54686
H	-3.38761	-4.69874	-3.29439
H	-1.50025	-4.15561	-5.0285
H	-0.19579	-4.51373	-3.90981
H	-0.27517	-2.91806	-4.65248
H	-3.82104	-1.45787	5.7728
H	-3.5734	-3.89092	6.07193
H	-2.96698	-5.31721	4.15416
H	-3.27347	0.37623	2.57041
H	-5.69845	-0.01459	2.9879
H	-5.18348	1.49889	3.73712
H	-5.42816	0.0615	4.73435
H	-3.04923	0.62243	5.62526
H	-2.8051	1.89624	4.43018
H	-1.65754	0.54808	4.54335
H	-2.37829	-4.10274	0.67605
H	-3.69515	-6.38735	2.24556
H	-3.36734	-6.33784	0.51353
H	-4.57977	-5.25188	1.21964
H	-0.32313	-4.65568	1.92676
H	-0.92843	-6.07294	1.0485
H	-1.16678	-5.95201	2.79292
H	-5.41313	-1.89551	2.17332
H	-7.63835	-2.90083	2.19749
H	-8.45852	-4.19732	0.2347
H	-7.0066	-4.4218	-1.77596
H	-4.79913	-3.39157	-1.82838
H	2.68987	-5.47983	-3.76022
H	3.31388	-4.13628	-5.7297
H	3.73198	-1.71975	-5.49494

H	2.3322	-4.17479	-0.28589
H	0.17012	-4.66946	-1.35051
H	0.79664	-6.10443	-0.52329
H	0.89542	-5.99712	-2.28145
H	3.41965	-6.55623	-1.88294
H	3.24459	-6.42873	-0.13206
H	4.44076	-5.43097	-0.97931
H	3.36725	0.2149	-2.32436
H	3.21424	0.4313	-5.38937
H	3.0953	1.74787	-4.2184
H	1.81134	0.52458	-4.33285
H	5.75631	-0.38887	-2.70631
H	5.3807	1.14783	-3.48562
H	5.50981	-0.32315	-4.45709
H	3.14981	-2.57524	5.68781
H	5.07597	-1.08159	6.04212
H	5.98845	0.22729	4.16301
H	1.33416	-3.01456	2.46091
H	1.01392	-3.0566	5.51811
H	-0.19181	-3.51192	4.31521
H	0.31501	-1.81944	4.47309
H	2.96731	-4.86081	2.80881
H	1.4185	-5.26811	3.55197
H	2.76613	-4.73529	4.56196
H	4.71077	0.14769	0.66067
H	4.05811	2.16982	1.91698
H	5.62144	2.42002	1.11686
H	5.56309	2.11188	2.85148
H	7.30804	0.24262	2.29249
H	7.12671	0.49928	0.55684

H	6.84753	-1.10875	1.24974
H	4.49974	-3.62542	2.11703
H	6.62087	-4.84446	2.13643
H	8.1682	-4.71014	0.19057
H	7.53717	-3.33434	-1.78663
H	5.40749	-2.12627	-1.82175
H	3.42826	4.89042	-3.8437
H	1.85678	4.952	-5.74197
H	-0.47306	4.24077	-5.44325
H	2.57485	3.79554	-0.38025
H	3.9138	2.16552	-1.65476
H	4.97494	3.29932	-0.80242
H	4.72366	3.47202	-2.53885
H	4.13689	5.94583	-1.91428
H	4.21755	5.59202	-0.18797
H	2.74254	6.2876	-0.88236
H	-1.85264	2.53967	-2.53787
H	-2.55781	3.85442	-5.21297
H	-3.40712	2.57134	-4.36497
H	-1.82912	2.23933	-5.09484
H	-2.30898	4.78737	-1.59019
H	-3.64629	4.31233	-2.63736
H	-2.44981	5.47095	-3.21904
H	0.49839	4.01768	5.73548
H	-1.76176	4.96628	5.97776
H	-3.25837	5.11092	4.02229
H	1.93412	2.6635	2.57447
H	1.98726	2.37498	5.63456
H	3.01386	1.54833	4.46316
H	1.28292	1.17244	4.55065

H	2.74103	4.97895	2.98772
H	3.79552	3.82157	3.80074
H	2.60931	4.7368	4.73579
H	-2.38528	4.00406	0.56839
H	-3.89426	2.47197	1.79101
H	-4.81741	3.67853	0.87252
H	-4.65069	3.8433	2.62086
H	-3.79038	6.26461	2.09522
H	-3.86425	5.94671	0.3615
H	-2.3449	6.48502	1.10095
H	0.9443	5.59701	2.196
H	1.15896	8.02447	2.28234
H	0.54123	9.41569	0.3092
H	-0.27255	8.30117	-1.76397
H	-0.46608	5.87796	-1.87269

References

1. N. K. T. Ho, B. Neumann, H.-G. Stammler, V. H. Menezes da Silva, D. G. Watanabe, A. A. C. Braga and R. S. Ghadwal, *Dalton Trans.*, 2017, **46**, 12027−12031.
2. Fulmer, G. R., Miller, A. J. M., Sherden, N. H., Gottlieb, H. E., Nudelman, A., Stoltz, B. M., Bercaw, J. E., Goldberg, K. I. *Organometallics* **2010**, *29*, 2176–2179.
3. Dolomanov, O. V., Bourhis, L. J., Gildea, R. J., Howard, J. A. K., Puschmann, H., OLEX2: a complete structure solution, refinement and analysis program, *J. Appl. Cryst.* **2009**, *42*, 339–341.
4. Sheldrick, G. M., A short history of SHELX, *Acta Cryst.* **2008**, A64, 112–122.
5. Sheldrick, G. M., Crystal structure refinement with SHELXL, *Acta Cryst.* **2015**, C71, 3–8.
6. Gaussian 16, Revision B.01, M. J. Frisch, G. W. Trucks, H. B. Schlegel, G. E. Scuseria, M. A. Robb, J. R. Cheeseman, G. Scalmani, V. Barone, G. A. Petersson, H. Nakatsuji, X. Li, M. Caricato, A. V. Marenich, J. Bloino, B. G. Janesko, R. Gomperts, B. Mennucci, H. P. Hratchian, J. V. Ortiz, A. F. Izmaylov, J. L. Sonnenberg, D. Williams-Young, F. Ding, F. Lipparini, F. Egidi, J. Goings, B. Peng, A. Petrone, T. Henderson, D. Ranasinghe, V. G. Zakrzewski, J. Gao, N. Rega, G. Zheng, W. Liang, M. Hada, M. Ehara, K. Toyota, R. Fukuda, J. Hasegawa, M. Ishida, T. Nakajima, Y. Honda, O. Kitao, H. Nakai, T. Vreven, K. Throssell, J. A. Montgomery, Jr., J. E. Peralta, F. Ogliaro, M. J. Bearpark, J. J. Heyd, E. N. Brothers, K. N. Kudin, V. N. Staroverov, T. A. Keith, R. Kobayashi, J. Normand, K. Raghavachari, A. P. Rendell, J. C. Burant, S. S. Iyengar, J. Tomasi, M. Cossi, J. M. Millam, M. Klene, C. Adamo, R. Cammi, J. W. Ochterski, R. L. Martin, K. Morokuma, O. Farkas, J. B. Foresman, and D. J. Fox, Gaussian, Inc., Wallingford CT, 2016.
7. (a) A.D. Becke, *J. Chem. Phys.*, **98** (1993) 5648-52; (b) S. H. Vosko, L. Wilk, and M. Nusair, *Can. J. Phys.*, **58** (1980) 1200-11; (c) C. Lee, W. Yang, and R. G. Parr, *Phys. Rev. B*, **37** (1988) 785-89; (d) P.J. Stephens, F.J. Devlin, C.F. Chabalowski and M.J. Frisch, *J. Phys. Chem.* **98** (1994) 11623-11627.
8. W. R. Wadt and P. J. Hay, *J. Chem. Phys.*, **82** (1985) 284-98.
9. R. Ditchfield, W. J. Hehre, and J. A. Pople *J. Chem. Phys.*, **54** (1971) 724.
10. Frisch, M. J., Trucks, G. W., Schlegel, H. B., Scuseria, G. E., Robb, M. A., Cheeseman, J. R., Scalmani, G., Barone, V., Mennucci, B., Petersson, G. A., Nakatsuji, H., Caricato, M., Li, X., Hratchian, H. P., Izmaylov, A. F., Bloino, J., Zheng, G., Sonnenberg, J. L., Hada, M., Ehara, M., Toyota, K., Fukuda, R., Hasegawa, J., Ishida, M., Nakajima, T., Honda, Y., Kitao, O., Nakai, H., Vreven, T., Montgomery, J. J. A., Peralta, J. E., Ogliaro, F., Bearpark, M., Heyd, J. J., Brothers, E., Kudin, K. N., Staroverov, V. N., Kobayashi, R., Normand, J., Raghavachari, K., Rendell, A., Burant, J. C., Iyengar, S. S., Tomasi, J., Cossi, M., Rega, N., Millam, J. M., Klene, M., Knox, J. E., Cross, J. B., Bakken, V., Adamo, C., Jaramillo, J., Gomperts, R.,

Stratmann, R. E., Yazyev, O., Austin, A. J., Cammi, R., Pomelli, C., Ochterski, J. W., Martin, R. L., Morokuma, K., Zakrzewski, V. G., Voth, G. A., Salvador, P., Dannenberg, J. J., Dapprich, S., Daniels, A. D., Farkas, O., Foresman, J. B., Ortiz, J. V., Cioslowski, J., Fox, D. J., Gaussian 16, Revision A.03. Gaussian, Inc., Wallingford CT, 2016.

11. Wiberg, K. B., *Tetrahedron* **1968**, *24*, 1083–1096.
12. (a) Reed, A. E., Weinhold, F., *J. Chem. Phys.* **1985**, *83*, 1736–1740; (b) Reed, A. E., Weinstock, R. B., Weinhold, F., *J. Chem. Phys.* **1985**, *83*, 735–746.
13. Glendening, E. D., Reed, A. E., Carpenter, J. E., Weinhold, F., NBO Version 3.1.
